# Tree mycorrhizal type regulates leaf and needle microbial communities, affects microbial assembly and co-occurrence network patterns, and influences litter decomposition rates in temperate forest

**DOI:** 10.3389/fpls.2023.1239600

**Published:** 2023-11-29

**Authors:** Benjawan Tanunchai, Li Ji, Simon Andreas Schroeter, Sara Fareed Mohamed Wahdan, Katikarn Thongsuk, Ines Hilke, Gerd Gleixner, François Buscot, Ernst-Detlef Schulze, Matthias Noll, Witoon Purahong

**Affiliations:** ^1^ Department of Soil Ecology, UFZ-Helmholtz Centre for Environmental Research, Halle (Saale), Germany; ^2^ Institute of Bioanalysis, Coburg University of Applied Sciences and Arts, Coburg, Germany; ^3^ Bayreuth Center of Ecology and Environmental Research (BayCEER), University of Bayreuth, Bayreuth, Germany; ^4^ School of Forestry, Central South of Forestry and Technology, Changsha, China; ^5^ Max Planck Institute for Biogeochemistry, Biogeochemical Processes Department, Jena, Germany; ^6^ Department of Botany and Microbiology, Faculty of Science, Suez Canal University, Ismailia, Egypt; ^7^ German Centre for Integrative Biodiversity Research (iDiv), Halle-Jena-Leipzig, Leipzig, Germany

**Keywords:** ecological drift, variable selection, N-fixing bacteria, enzyme activity, arbuscular mycorrhiza, ectomycorrhiza

## Abstract

**Background:**

Tree mycorrhizal types (arbuscular mycorrhizal fungi and ectomycorrhizal fungi) alter nutrient use traits and leaf physicochemical properties and, thus, affect leaf litter decomposition. However, little is known about how different tree mycorrhizal species affect the microbial diversity, community composition, function, and community assembly processes that govern leaf litter-dwelling microbes during leaf litter decomposition.

**Methods:**

In this study, we investigated the microbial diversity, community dynamics, and community assembly processes of nine temperate tree species using high-resolution molecular technique (Illumina sequencing), including broadleaved arbuscular mycorrhizal, broadleaved ectomycorrhizal, and coniferous ectomycorrhizal tree types, during leaf litter decomposition.

**Results and discussion:**

The leaves and needles of different tree mycorrhizal types significantly affected the microbial richness and community composition during leaf litter decomposition. Leaf litter mass loss was related to higher sequence reads of a few bacterial functional groups, particularly N-fixing bacteria. Furthermore, a link between bacterial and fungal community composition and hydrolytic and/or oxidative enzyme activity was found. The microbial communities in the leaf litter of different tree mycorrhizal types were governed by different proportions of determinism and stochasticity, which changed throughout litter decomposition. Specifically, determinism (mainly variable selection) controlling bacterial community composition increased over time. In contrast, stochasticity (mainly ecological drift) increasingly governed fungal community composition. Finally, the co-occurrence network analysis showed greater competition between bacteria and fungi in the early stages of litter decomposition and revealed a contrasting pattern between mycorrhizal types.

**Conclusion:**

Overall, we conclude that tree mycorrhizal types influence leaf litter quality, which affects microbial richness and community composition, and thus, leaf litter decomposition.

## Introduction

The decomposition of plant litter plays a crucial role in regulating carbon (C) and nutrient cycles in terrestrial forest ecosystems (Aerts and de Caluwe, 1997; Hobbie, 2015). Leaf litter highly contributes to the detritusphere, an interphase between the above- and belowground in forest ecosystems where intensive interactions among microbes occur (Ma et al., 2017). Leaf litter decomposition rates determine the velocity of nutrient turnover and transfer from primary producers to other organisms (Keller and Phillips, 2019). Thus, litter decomposition contributes significantly to nutrient availability, soil fertility, and productivity of terrestrial forest ecosystems (Aerts and de Caluwe, 1997; Hobbie, 2015). Litter decomposition is controlled by both abiotic factors, such as climate, environmental factors, and physicochemical properties of the litter, and biotic factors, especially cross-kingdom interactions between soil bacteria and fungi (Berg, 2000; Purahong et al., 2016). Leaf litter decomposition in forest ecosystems can even be influenced by soil microbes before leaf senescence has begun, through the symbiosis between host trees and mycorrhizal fungi (Jacobs et al., 2018; Keller and Phillips, 2019; Seyfried et al., 2021). Forest trees are associated with two dominant types of fungi, namely, arbuscular mycorrhizal (AM) and ectomycorrhizal (EcM) fungi, to improve their nutrient uptake, growth, and fitness (Bonfante and Genre, 2010). These different types of symbioses between AM and EcM trees have been demonstrated to alter nutrient use traits that significantly affect leaf physicochemical properties and quality (Keller and Phillips, 2019; Seyfried et al., 2021). Specifically, EcM trees tend to produce lower quality leaf litter than AM trees (Seyfried et al., 2021). In temperate forests, such differences in leaf quality have been reported to affect leaf litter decomposition rates, which are usually higher in AM trees than in EcM trees (Keller and Phillips, 2019). Leaf litter nitrogen (N) content and tree phylogeny have been identified as significant factors that explain the difference in litter decomposition rates between AM and EcM trees (Keller and Phillips, 2019). Furthermore, competition between EcM fungi and saprotrophs in EcM-dominated forests can negatively affect litter decomposition in EcM trees (Seyfried et al., 2021). However, little is known about how different tree mycorrhizal types affect microbial diversity, community composition, and function during leaf litter decomposition. The mechanisms underlying the assembly of microbial communities and their cross-kingdom interactions in plant litter of AM and EcM trees remain largely unexplored (Purahong et al., 2016; Abrego, 2021). Several studies have shown that fungal community assembly in temperate forests is governed by stochastic processes (dispersal limitation and drift), whereas both stochastic and deterministic processes dominate the bacterial community assembly (Osburn et al., 2021; Zhang et al., 2022). However, this information may not be fully applicable to microbes living in leaf litter, and the relative importance of each specific assembly process may vary greatly between the leaf litter of different tree mycorrhizal types and decomposition stages.

Cross-kingdom interactions, particularly between bacteria and fungi, are the main drivers of plant litter decomposition (Purahong et al., 2016). However, the dynamics of cross-kingdom interactions during litter decomposition and among different mycorrhizal types have not yet been investigated. While both bacteria and fungi play an important role as direct decomposers through the production and secretion of extracellular plant compound-degrading enzymes, different functional groups of bacteria act as facilitators by providing additional macronutrients such as N and phosphorus (P) for fungal decomposers (Purahong et al., 2016; Mieszkin et al., 2021; Purahong et al., 2022). Tree mycorrhizal type can alter the abundance and microbial community composition of leaf litter-dwelling microbes due to its significant impact on soil microbial communities (Singavarapu et al., 2021) and leaf litter properties (Seyfried et al., 2021). Thus, the key microbial players in leaf litter decomposition, as well as their interactions with biotic and abiotic factors, may differ greatly among the tree mycorrhizal types. However, this has not been tested yet.

The objectives of this study were to i) investigate microbial diversity, community composition of leaves and needles, and environmental factors in nine tree species representing different tree mycorrhizal types; ii) investigate microbial community assembly over time; iii) investigate the dynamics of co-occurrence network patterns of different tree mycorrhizal types over time and identify their associated keystone microbial taxa; and vi) investigate the relationship between microbial functions (enzyme activities) and microbial communities. We hypothesized that i) tree mycorrhizal types determine microbial richness and community composition through their specific initial physicochemical properties of the leaves and needles and ii) microbial community assembly differs among different tree mycorrhizal types and over time. We expected different co-occurrence network patterns and their associated keystone microbial taxa over time and among different tree mycorrhizal types.

## Materials and methods

### Study site, experimental setup, and design

The leaf litter decomposition experiment was conducted at the study site located in the Hainich-Dün region of Thuringia, Germany (51°12'N, 10°18'E). Mature leaves and needles were collected, oven-dried at 25°C for 14 days, and returned under their mother tree. Further details of the experimental design and study site have been published elsewhere (Tanunchai et al., 2022a) and in the **Supplementary Material**.

In October 2019, at least 200 g of mature leaves and needles were collected from three tree mycorrhizal types, each of which was represented by three tree species. Five true tree replicates were collected for each tree species, at a minimum of 5 m apart (45 trees in total). The tree mycorrhizal types investigated in this study were i) broadleaved arbuscular mycorrhizal trees (AM_BL; *Acer pseudoplatanus*, *Fraxinus excelsior*, and *Prunus avium*), ii) broadleaved ectomycorrhizal trees (EcM_BL; *Fagus sylvatica*, *Carpinus betulus*, and *Tilia cordata*), and iii) coniferous ectomycorrhizal trees (EcM_C; *Picea abies*, *Pinus sylvestris*, and *Pseudotsuga menziesii*). The collected mature leaves and needles were oven-dried at 25°C for 14 days. A nylon bag (2 mm mesh, 5 mm holes) was filled with 3 g of oven-dried leaves and needles. The nylon bags were then placed under the same mother tree to mimic the actual situation of leaf litter decomposition in the environment. After 200 and 400 days of decomposition, leaf/needle samples were collected in separate sterile plastic bags with new clean gloves, transported on ice to the laboratory within 3 h, and stored at −80°C for further analysis. Another set of samples was sent on ice to the physicochemical laboratory to determine leaf/needle water content, pH, and nutrients. All further analyses are summarized in the experimental scheme (**Supplementary Material**).

### Physiochemical and enzyme analyses

The procedures for the physicochemical analyses were published by Tanunchai et al. (2022a). Total leaf C (C), total leaf N (N), nutrient (Ca, Fe, K, Mg, and P contents), dissolved organic C (DOC), dissolved organic N (N_org_), and dissolved inorganic N (N_min_) contents were analyzed. Leachable components were extracted by incubating wet leaf and needle samples in 30 mL of MilliQ water for 1 h at room temperature. Five potential enzymatic activities, including three hydrolytic enzymes (β-glucosidase, N-acetylglucosaminidase, and acid phosphatase) and two oxidative enzymes (general peroxidase and manganese peroxidase) (Purahong et al., 2016), were measured in homogenized leaves and needles. More details on the physicochemical and enzymatic analyses are provided in the **Supplementary Material**.

### DNA extraction and Illumina sequencing

The procedures for DNA extraction, Illumina sequencing, and bioinformatics have been published by Tanunchai et al. (2022a). Further details are provided in the **Supplementary Material**. Briefly, leaf and needle samples were washed three times in sterile Tween solution (0.1% vol/vol), washed three to five times using deionized water, and then incubated for 1 h in sterile water. The ground leaf samples (~120 mg homogenized leaves and needles) were subjected to DNA extraction using the DNeasy PowerSoil Kit (Qiagen, Hilden, Germany) and a Precellys 24 tissue homogenizer (Bertin Instruments, Montigny-le-Bretonneux, France) according to the manufacturer’s instructions.

The microbial communities associated with leaves and needles were profiled by amplification and sequencing of two genetic markers: the fungal internal transcribed spacer 2 (ITS2) within the nuclear ribosomal DNA (rDNA) and the bacterial 16S rRNA gene V4 region (Weißbecker et al., 2020). The fungal ITS2 region was amplified using the fungal universal primer pair fITS7 [5'-GTGARTCATCGAATCTTTG-3'] (Ihrmark et al., 2012) and ITS4 primer [5'-TCCTCCGCTTATTGATATGC-3'] (White et al., 1990) with Illumina adapter sequences. The 16S rRNA gene V4 region was amplified using the universal bacterial primer pair 515F (5'-GTGCCAGCMGCCGCGGTAA-3') and 806R (5'-GGACTACHVGGGTWTCTAAT-3') (Caporaso et al., 2011) with Illumina adapter sequences. Paired-end sequencing (2 × 300 bp) was performed on the pooled PCR products using a MiSeq Reagent kit v3 on an Illumina MiSeq system (Illumina Inc., San Diego, CA, USA) at the Department of Soil Ecology, Helmholtz Centre for Environmental Research, Germany.

### Bioinformatics

The 16S rRNA and ITS2 gene sequences corresponding to the forward and reverse primers were trimmed from the demultiplexed raw reads using Cutadapt (Martin, 2011). The paired-end sequences were quality-trimmed, filtered for chimeras, and assembled using the DADA2 package (Callahan et al., 2016) through the pipeline dadasnake (Weißbecker et al., 2020). Assembled reads that met these criteria were retained for further analysis. High-quality reads were clustered into 15,213 bacterial and 5,030 fungal amplicon sequence variants (ASVs) after chimera removal. Rare ASVs (singletons) were removed as they may represent artificial sequences. The datasets were then rarefied to the minimum sequencing depth of bacterial and fungal sequence reads (21,000 sequences per sample). The bacterial sequencing data of mature leaves and needles (at 0 days) were not considered for rarefaction because their minimum sequence reads were 2,733 reads, which is approximately eight times lower than the minimum reads of the total samples. The richness at the different rarefaction depths of these samples was determined (**Table S1**). Finally, 14,773 rarefied bacteria and 4,896 fungal ASVs were obtained. Using absolute sequence reads instead of relative sequence read abundances reflects to a higher degree the PCR pitfalls, as reviewed earlier (Wintzingerode et al., 1997); therefore, the use of relative sequence read abundances is recommended (Schloss et al., 2009). To avoid sequencing bias, normalizing the data by rarefaction is recommended. After rarefaction to a minimum of 21,000 sequence reads, both bacterial and fungal rarefaction curves showed saturation, which implies that a large majority of the microbes in the community were included. The rarefaction curves of all samples reached saturation (**Supplementary Material**), which is a prerequisite for less biased sequence comparisons between samples (Schloss et al., 2009). Nevertheless, it should be noted that the rarefied data usually differ from the raw sequence read data, which may lead to a different pattern in the analyzed results, which in turn may affect ecological interpretation. Thus, the Mantel test based on the Bray–Curtis distance with 999 permutations was applied to evaluate the correlation between the whole matrix and a rarified matrix for bacterial and fungal datasets (Tanunchai et al., 2022b). The results indicated that the rarefaction dataset was highly representative of the entire bacterial and fungal matrices (*R*_Mantel, bacteria_ = 0.998, *P* = 0.001; *R*_Mantel, fungi_ = 0.996, *P* = 0.001). Datasets of relative sequence read abundance were used for statistical analyses. It is also important to note that sequencing data provide only information regarding the occurrence and relative abundance of taxa, but it is not a direct measure of the absolute abundance of the taxa in the samples. To approximate the absolute abundances, further methods, such as the incorporation of internal standards of known quantity (Harrison et al., 2021) or quantitative PCR (Tanunchai et al., 2023), should be considered. The metabolic functional profiles of leaf-associated bacterial communities in nine temperate tree species were predicted using Tax4Fun2 in R (v4.0.5) (Wemheuer et al., 2020, 2). The fungal ecological function of each ASV was determined using FungalTraits (Põlme et al., 2020; Tanunchai et al., 2022a), according to the authors’ instructions. Further details are provided in the **Supplementary Material**.

### Network and community assembly analyses

Based on the random matrix theory (RMT), we constructed co-occurrence networks of cross-kingdoms inhabiting mature and decomposing leaves using the molecular ecological network analysis pipeline (MENA, http://ieg4.rccc.ou.edu/mena/). The network analysis was performed following the four steps described in previous studies (Zhou et al., 2011; Deng et al., 2012): 1) amplification sequence read collection, 2) data standardization, 3) pairwise similarity estimation, and 4) adjacent matrix construction according to an RMT-based approach. Further details are provided in the **Supplementary Material**. The phylogenetic normalized stochasticity ratio (pNST) based on the null model theory was used to quantify the relative proportion of deterministic and stochastic processes in community assembly. All networks were visualized with Gephi v0.9.2. All of the parameters were calculated using the “iCAMP” package in R with the code provided by Ning et al. (2020) (https://github.com/DaliangNing/iCAMP1).

### Statistical analysis

The datasets were tested for normality using the Jarque–Bera test and for equality of group variances using the *F*-test (for two datasets) and Levene’s test (for more than two datasets). The effects of time, tree species, and tree mycorrhizal type on microbial community composition were visualized using non-metric multidimensional scaling (NMDS) and tested using analysis of similarities (ANOSIM) and non-parametric multivariate analysis of variance (NPMANOVA) based on relative abundance data and the Bray–Curtis distance measure. Over 999 permutations were performed. The relationship between different environmental factors, enzyme activities, and microbial community composition was analyzed using a goodness-of-fit statistic based on normalized relative abundance and the Bray–Curtis distance measure. The effects of time, tree species, and tree mycorrhizal type on leaf litter mass loss, microbial ASV richness, leaf physicochemical properties, and enzyme activities were tested using repeated measures analysis of variance (ANOVA) with Fisher’s least significant difference (LSD) *post-hoc* test. Log transformation was used when necessary. All statistical analyses were performed using the PAST version 2.17, SPSS version 29.0, R, and RStudio version 4.2.1.

## Results

### Microbial succession during leaf litter decomposition

Details of the general overview of the leaf and needle microbiomes in forest ecosystems are provided in the **Supplementary Material**. We found three patterns of microbial succession over decomposition time. First, some microbes were enriched at 200 and 400 days of decomposition and were not initially detected in mature leaves and needles. These microbes included bacteria (such as *Caulobacter* (ASV10), *Flavobacterium* (ASV38), *Brevundimonas* (ASV44), *Rhizobiaceae* (ASV48), *Polaromonas* (ASV51, and ASV54); **Figures S1, S2**; **Table S2**) and fungi (such as *Botryosphaeriales* (ASV12), *Herpotrichia* (ASV30), *Chaetomium* (ASV8, and ASV19); **Figures S3, S4**; **Table S3**). While the enrichment of such bacteria at 200 and 400 days was consistent across all tree mycorrhizal types, the enrichment of fungi was more specific to some tree mycorrhizal types. *Botryosphaeriales* (fungal ASV12) was enriched only on needles of the EcM_C tree, specifically of *P. menziesii* (**Figures S3, S4**). *Chaetomium* spp. were enriched in the leaves of AM_BL (*P. avium*) and EcM_BL trees (*T. cordata*, **Figures S3, S4**). Second, the relative sequence read abundances of bacteria (*Sphingomonas* (ASV6), *Massilia* (ASV11)) and fungi (*Helotiales* (ASV10), *Aureobasidium* (ASV9), *Mycosphaerellaceae* (ASV31), and *Didymellaceae* (ASV24)) were reduced at 200 and 400 days (**Figures S1–S4**). The majority of these taxa were initially highly enriched in the leaves of both AM_BL and EcM_BL trees. Third, some microbes were highly enriched at 200 days of decomposition. These microbes include bacteria (*Pseudomonas* (ASV4), *Pedobacter* (ASV9), *Microbacteriaceae* (ASV8), and *Luteibacter* (ASV17)) and fungi (*Alternaria* (ASV2), *Tetracladium* (ASV14), and *Mollisina* (ASV17)). While the enrichment of such bacteria at 200 days was consistent across all tree mycorrhizal types (especially for EcM_C trees), the enrichment of fungi was specific to AM_BL and/or EcM_BL trees (**Figures S1–S4**). Interestingly, *Phoma* (fungal ASV7) was highly enriched in *P. sylvestris* needles at 400 days of decomposition (**Figure S4**).

The absolute number of reads was checked to validate the interpretation of the relative abundance data. Similar patterns were observed in this study. First, bacteria (*Caulobacter* (ASV10), *Flavobacterium* (ASV38), *Brevundimonas* (ASV44), *Rhizobiaceae* (ASV48), *Polaromonas* (ASV51), *Polaromonas* (ASV54)) and fungi (*Botryosphaeriales* (ASV12), *Herpotrichia* (ASV30), *Chaetomium* (ASV8, and ASV19)) were initially not detected but were enriched at 200 and 400 days of decomposition (**Table S4**). Second, the absolute number of reads of bacteria, including *Sphingomonas* (ASV6), *Massilia* (ASV11), and fungi, including *Helotiales* (ASV10), *Aureobasidium* (ASV9), *Mycosphaerellaceae* (ASV31), and *Didymellaceae* (ASV24) declined mainly after 400 days (**Table S4**). Third, some bacteria such as *Pseudomonas* (ASV4), *Pedobacter* (ASV9), *Microbacteriaceae* (ASV8), *Luteibacter* (ASV17) and fungi such as *Alternaria* (ASV2), *Tetracladium* (ASV14), and *Mollisina* (ASV17) were also enriched at 200 days of decomposition (**Table S4**).

### Tree species and mycorrhizal types drive changes in the microbial communities, ASV richness, and thus, leaf litter decomposition over time

**Figure 1:**
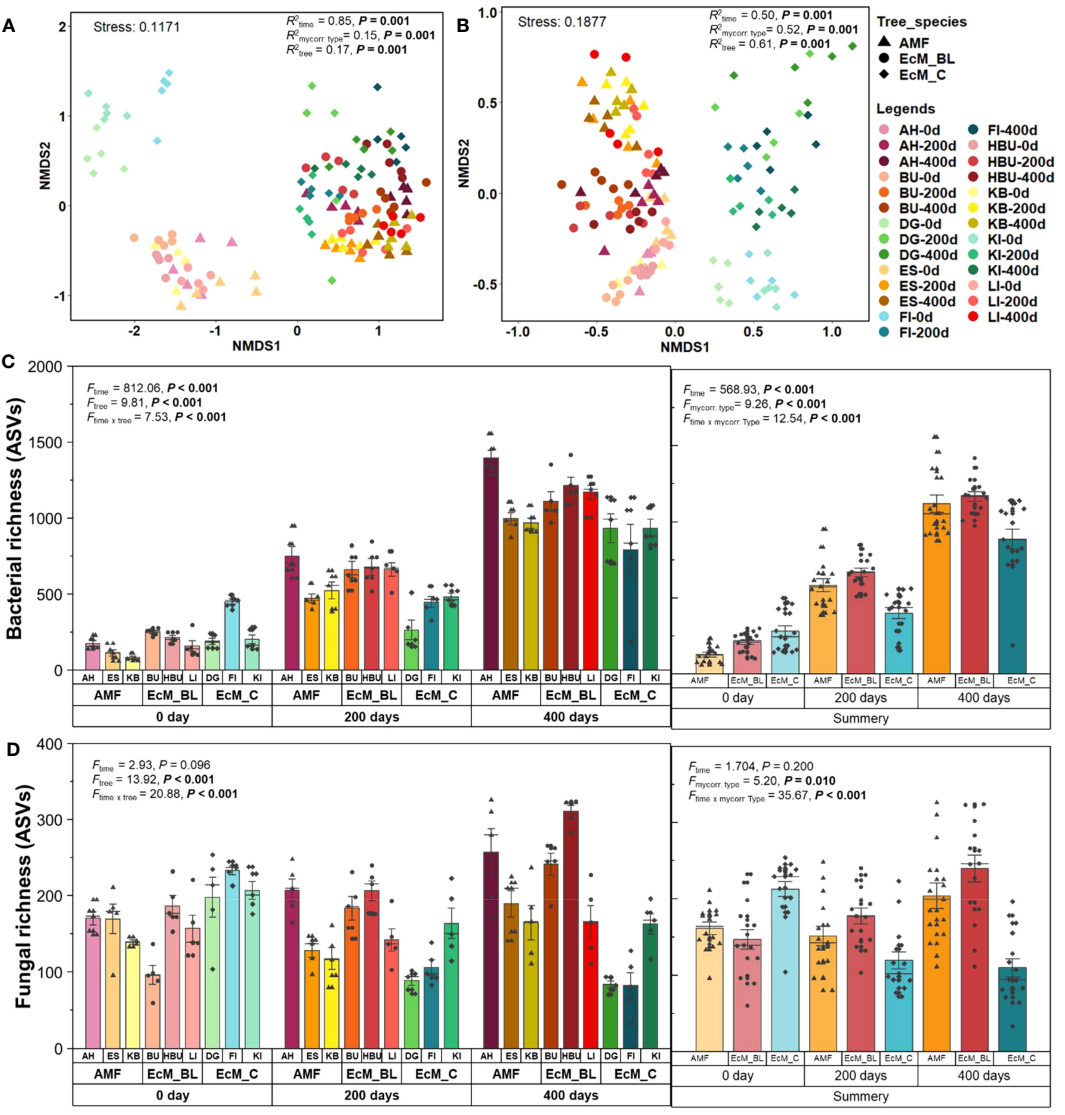
Non‐metric multidimensional scaling (NMDS) ordinations of bacterial **(A)** and fungal **(B)** community compositions in leaf and needle decomposition based on relative abundance. ASV richness (number of ASVs) for bacteria **(C)** and fungi **(D)** in leaf and needle decomposition. The legends for each NMDS data point are provided in the upper right of the figure. AM_BL, broadleaved arbuscular mycorrhizal trees (including AH, *Acer pseudoplatanus*; ES, *Fraxinus excelsior*; and KB, *Prunus avium*); EcM_BL, broadleaved ectomycorrhizal trees (including BU: *Fagus sylvatica*, HBU: *Carpinus betulus*, and LI: *Tilia cordata*); EcM_C, coniferous ectomycorrhizal trees (including FI, *Picea abies*; KI, *Pinus sylvestris*; and DG, *Pseudotsuga menziesii*). The results of PERMANOVA and ANOSIM are presented in **Table S5**. Statistical differences between microbial ASV richness among different tree species and tree mycorrhizal types were tested using repeated measures analysis of variance (ANOVA) with Fisher’s least significant difference (LSD) *post-hoc* test.

The microbial community composition in mature and decomposing leaves and needles differed among tree species, mycorrhizal types, and decomposition times (bacteria: *R*_ANOSIM_ = 0.94, *F*_NPMANOVA_ = 11.46, *P* < 0.001; fungi: *R*_ANOSIM_ = 0.95, *F*_NPMANOVA_ = 7.54, *P* < 0.001; **Figure 1**; **Table S5**). Tree species, tree mycorrhizal types, and sampling time significantly influenced the bacterial and fungal community composition across all sampling times (bacteria: *R* ^2^ = 0.15–0.94, *P* < 0.001; fungi: *R* ^2^ = 0.50–0.97, *P* < 0.001, **Table 1**). While tree mycorrhizal type was detected as the main factor determining the bacterial and fungal community composition only at 0 days (*R* ^2^ = 0.77–0.88, *P* < 0.001), tree species was the main factor controlling the microbial community composition at all sampling times (*R* ^2^ = 0.87–0.97, *P* < 0.001, **Table 1**). Sampling time was also the main factor that significantly altered bacterial community composition (*R* ^2^ = 0.85, *P* < 0.001, **Table 1**).

**Table 1:** Goodness-of-fit statistics (*R* ^2^) of environmental variables fitted to the non-metric multidimensional scaling (NMDS) ordination of bacterial and fungal communities in all tree species based on relative abundance data and Bray–Curtis distance measure.

Factors	All sampling times		0 days		200 days		400 days	
	Bacteria	Fungi	Bacteria	Fungi	Bacteria	Fungi	Bacteria	Fungi
Tree factors								
Tree mycorrhizal type	0.15***	0.52***	0.77***	0.88***	0.59***	0.61***	0.62***	0.64***
Tree species	0.17***	0.61***	0.94***	0.97***	0.87***	0.91***	0.90***	0.94***
Time factor								
Sampling times	0.85***	0.50***	NA	NA	NA	NA	NA	NA
Plot factors								
Soil water content	0.23***	0.43***	0.41***	0.45***	0.65***	0.64***	0.37***	0.43***
Soil pH	0.14**	0.04	0.44***	0.40***	0.33***	0.17*	0.27**	0.15*
Latitude	0.42***	0.58***	0.63***	0.70***	0.47***	0.61***	0.65***	0.64***
Longitude	0.10**	0.17***	0.15*	0.12	0.05	0.35***	0.18*	0.40***
Leaf physicochemical properties								
Leaf water content	0.16***	0.22***	0.22**	0.18*	0.49***	0.42***	0.40***	0.39***
Leaf pH	0.36***	0.30***	0.19*	0.27**	0.45***	0.30***	0.28***	0.36***
C content	0.41***	0.52***	0.72***	0.55***	0.49***	0.58***	0.37***	0.34**
DOC content	0.60***	0.25***	0.62***	0.56***	0.15*	0.06	0.11	0.07
N content	0.37***	0.19***	0.17*	0.16*	0.09	0.07	0.03	0.17*
N_min_ content	0.10**	0.06*	0.34**	0.23**	0.08	0.07	0.02	0.04
N_org_ content	0.37***	0.07**	0.60***	0.46***	0.23**	0.03	0.04	0.03
C:N ratio	0.57***	0.35***	0.17*	0.12	0.23**	0.21**	0.13	0.27**
C:P ratio	0.12***	0.13***	0.20*	0.35***	0.08	0.07	0.25**	0.20**
N:P ratio	0.19***	0.21***	0.66***	0.67***	0.16*	0.15*	0.26**	0.22**
Ca concentration	0.36***	0.39***	0.66***	0.60***	0.40***	0.38***	0.33***	0.26**
Fe concentration	0.29***	0.24***	0.25**	0.26**	0.23**	0.34***	0.34***	0.21*
K concentration	0.06*	0.06*	0.03	0.02	0.04	0.19*	0.18*	0.08
Mg concentration	0.23***	0.26***	0.48***	0.43***	0.37***	0.43***	0.17*	0.15*
P concentration	0.25***	0.17***	0.35***	0.38***	0.11	0.09	0.39***	0.35***

Bold values indicate statistical significance with *R* ^2^ ≥ 0.70.

*P < 0.05, **P < 0.01, ***P < 0.001, NA = Not applicable..

Tree mycorrhizal types also significantly affected microbial richness throughout the decomposition period (**Table S6**; **Figure 1**). At 0 day, microbial richness was the highest in the needles of EcM_C trees. In addition, we found that decomposition time significantly affected bacterial richness. The bacterial richness in the leaves and needles of all mycorrhizal types increased significantly with time (**Figure 1**; **Table S6**). However, no significant effect of decomposition time on fungal richness was observed (**Table S6**). Fungal richness observed in the leaves of AM_BL and EcM_BL trees tended to increase over time, while the fungal richness in the needles of EcM_C trees decreased after 200 days of incubation.

**Figure 2:**
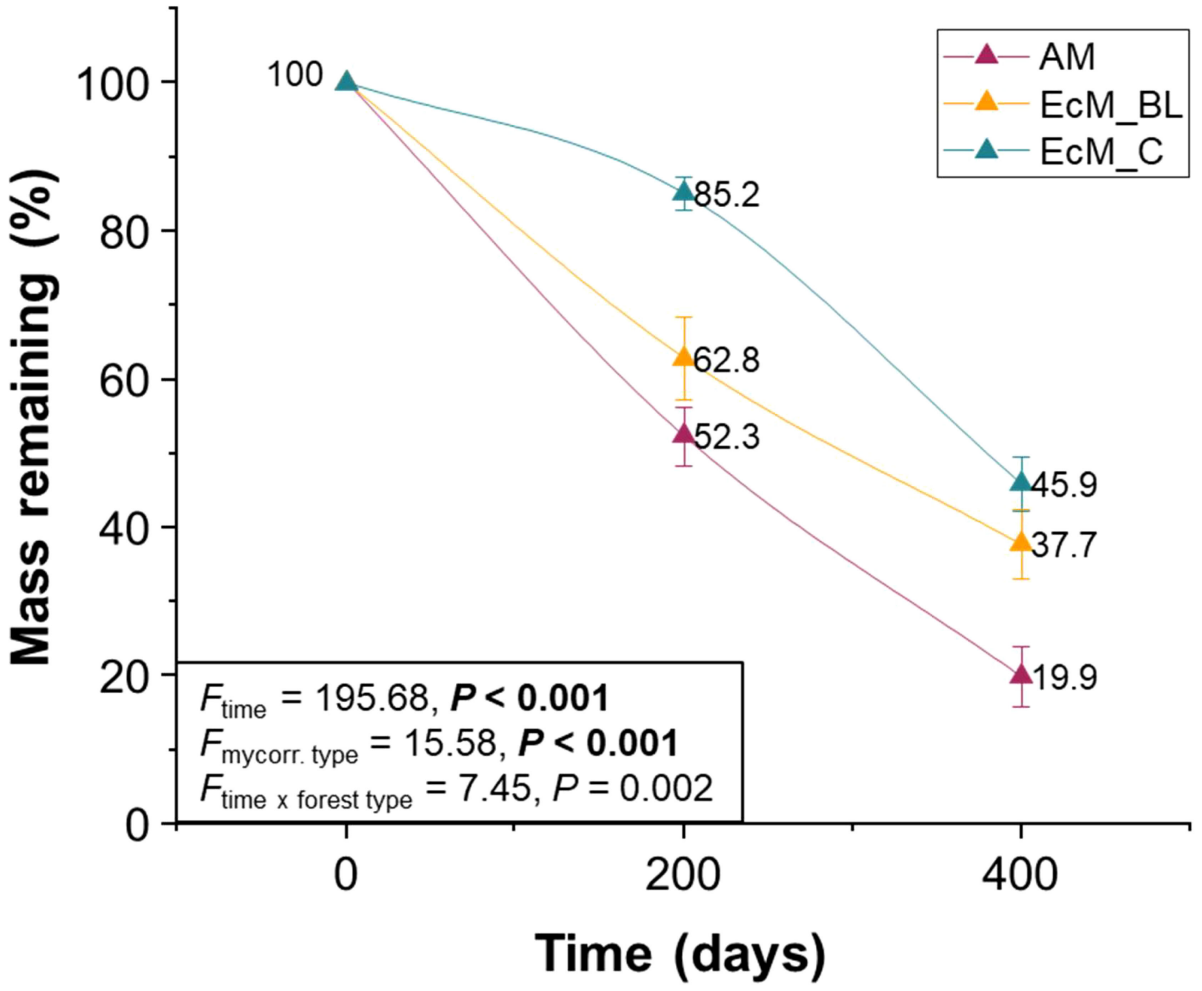
Molar mass loss of the leaves and needles of nine temperate tree species after 200 and 400 days of exposure. AM_BL, broadleaved arbuscular mycorrhizal trees; EcM_BL, broadleaved ectomycorrhizal trees; EcM_C, coniferous ectomycorrhizal trees. Statistical differences between molar mass losses among different tree species were tested using repeated measures analysis of variance (ANOVA) with Fisher’s least significant difference (LSD) *post-hoc* test.

The tree mycorrhizal type and decomposition time significantly influenced the mass loss of the leaves and needles (**Figure 2**). Mass losses were the lowest in ectomycorrhizal conifers [at 200 days, values ranged from 14.8% ± 2.2% (mean ± SE), and at 400 days, values ranged from 54.1% ± 3.6% (mean ± SE)] and the highest in AM_BL trees [at 200 days, values ranged from 47.7% ± 4.0% (mean ± SE), and at 400 days, values ranged from 80.1% ± 4.1% (mean ± SE)] (**Figure 2**). 

### Different assembly patterns in litter-associated bacterial and fungal communities across tree mycorrhizal types

**Figure 3:**
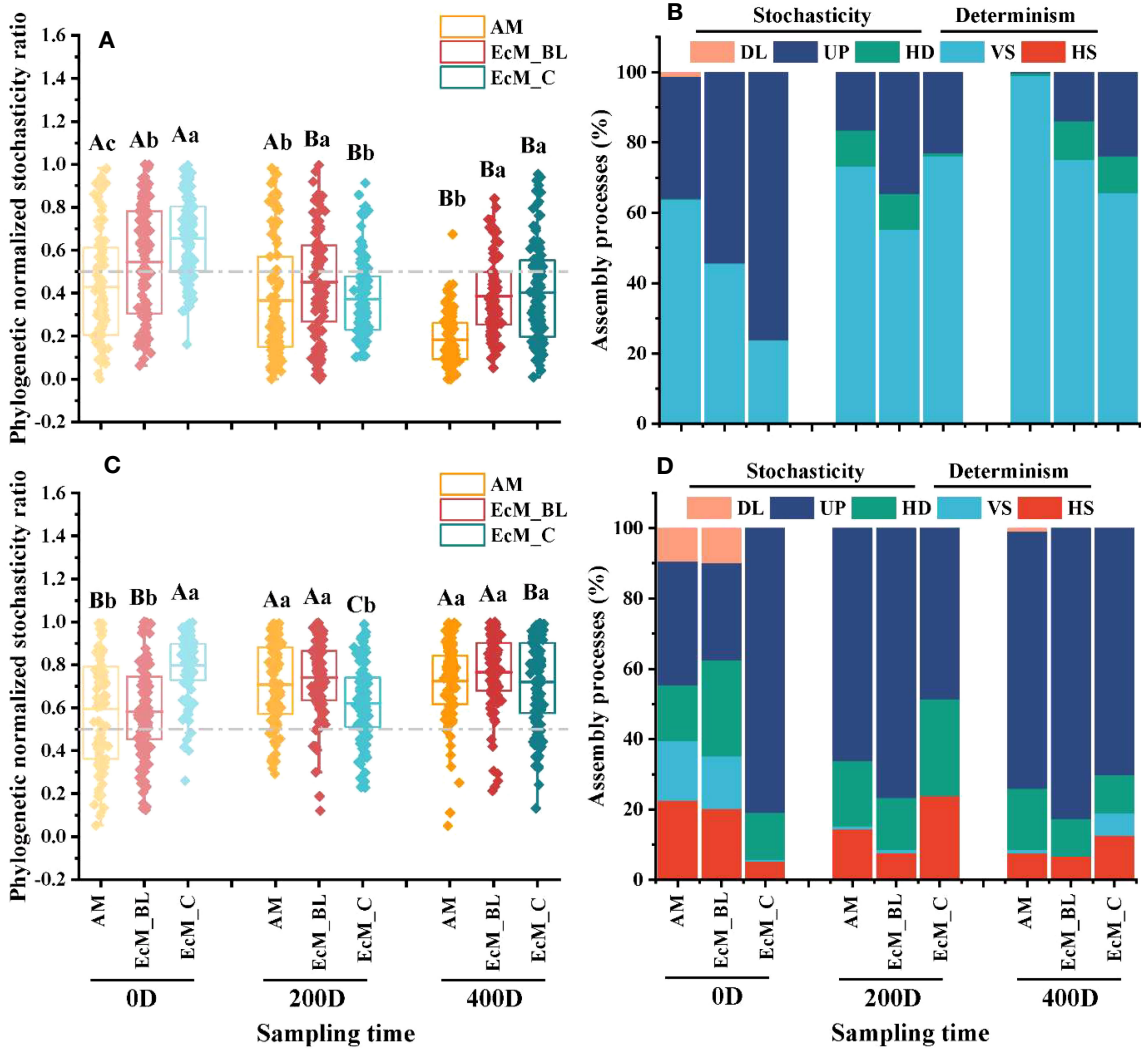
The ecological stochasticity in the potentially litter-associated bacterial **(A)** and fungal **(C)** community assembly estimated by the phylogenetic normalized stochasticity ratio (pNST). The value of 0.5 represents the boundary between the more deterministic (<0.5) and more stochastic (>0.5) assemblies. Data with different capital letters indicate significant differences at the 5% level between different sampling times in the same mycorrhizal type (*P* < 0.05), while different lowercase letters indicate significant differences between different mycorrhizal types in the same sampling time (*P* < 0.05). The relative contributions (%) of the community assembly processes based on pNST in shaping the litter-associated bacterial **(B)** and fungal **(D)** communities. HS, homogeneous selection; VS, variable selection; HD, homogenizing dispersal; UP, undominated process; DL, dispersal limitation; AM_BL, broadleaved arbuscular mycorrhizal trees; EcM_BL, broadleaved ectomycorrhizal trees; EcM_C, coniferous ectomycorrhizal trees.

Based on the null model, the relative contribution of ecological stochasticity to bacterial and fungal community assembly was calculated. The pNST values of the litter-associated bacterial communities of all trees at 400 days were lower than those at 0 days, and the relative proportion of variable selection increased over time (**Figures 3A, B**, *P* < 0.05). Compared with AM_BL trees, the bacterial communities inhabiting EcM_BL and/ or EcM_C trees had a higher pNST value (*P* < 0.05), suggesting that fewer stochastic processes were observed in AM_BL trees. For fungi, the pNST values of the AM_BL and EcM_BL trees increased with decomposition time (**Figure 3C**, *P* < 0.05). In contrast, the pNST value of EcM_C trees at 200 days was the lowest.

At 200 and 400 days of decomposition, the community assembly of litter-associated bacteria was mainly controlled by variable selection processes, whereas the fungal community was governed by drift (**Figures 3B, D**). In addition, a higher proportion of drift in the litter-associated bacterial community was found in the EcM_C trees at 0 day (**Figure 3B**). More importantly, a higher drift process of the litter-associated fungal community was observed at 200 and 400 days (**Figure 3D**).

### The microbial co-occurrence network and keystone taxa are important for leaf and needle decomposition

**Figure 4:**
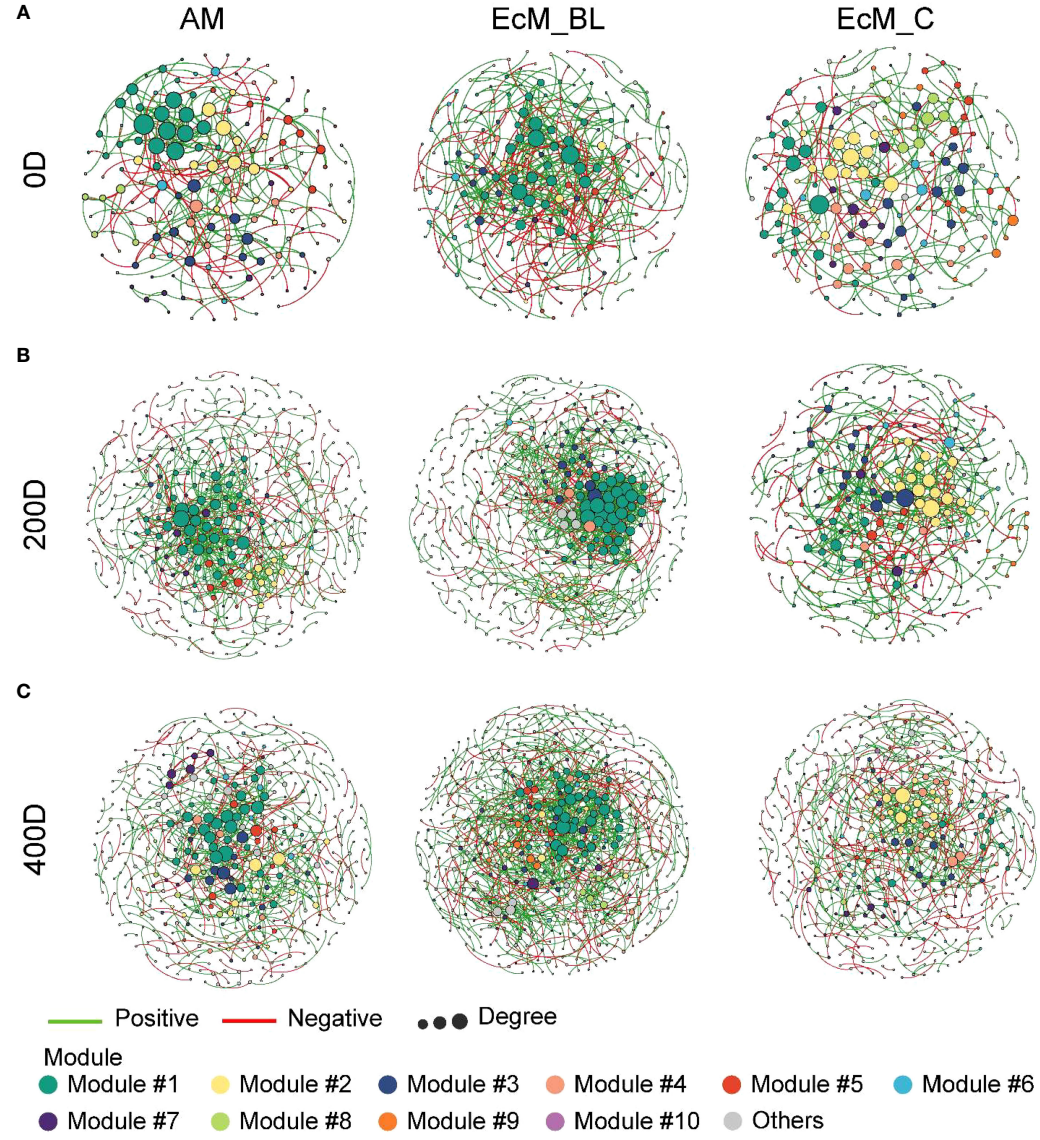
Interkingdom modular networks among mycorrhizal types and sampling times. The node colors represent different modules. **(A)** Initial litter phase (0 days); **(B)** 200 days of litter decomposition; **(C)** 400 days of litter decomposition. The connections denote a strong (Spearman’s *ρ* > 0.6) and significant (*P* < 0.01) correlations. AM_BL, broadleaved arbuscular mycorrhizal trees; EcM_BL, broadleaved ectomycorrhizal trees; EcM_C, coniferous ectomycorrhizal trees.

**Figure 5:**
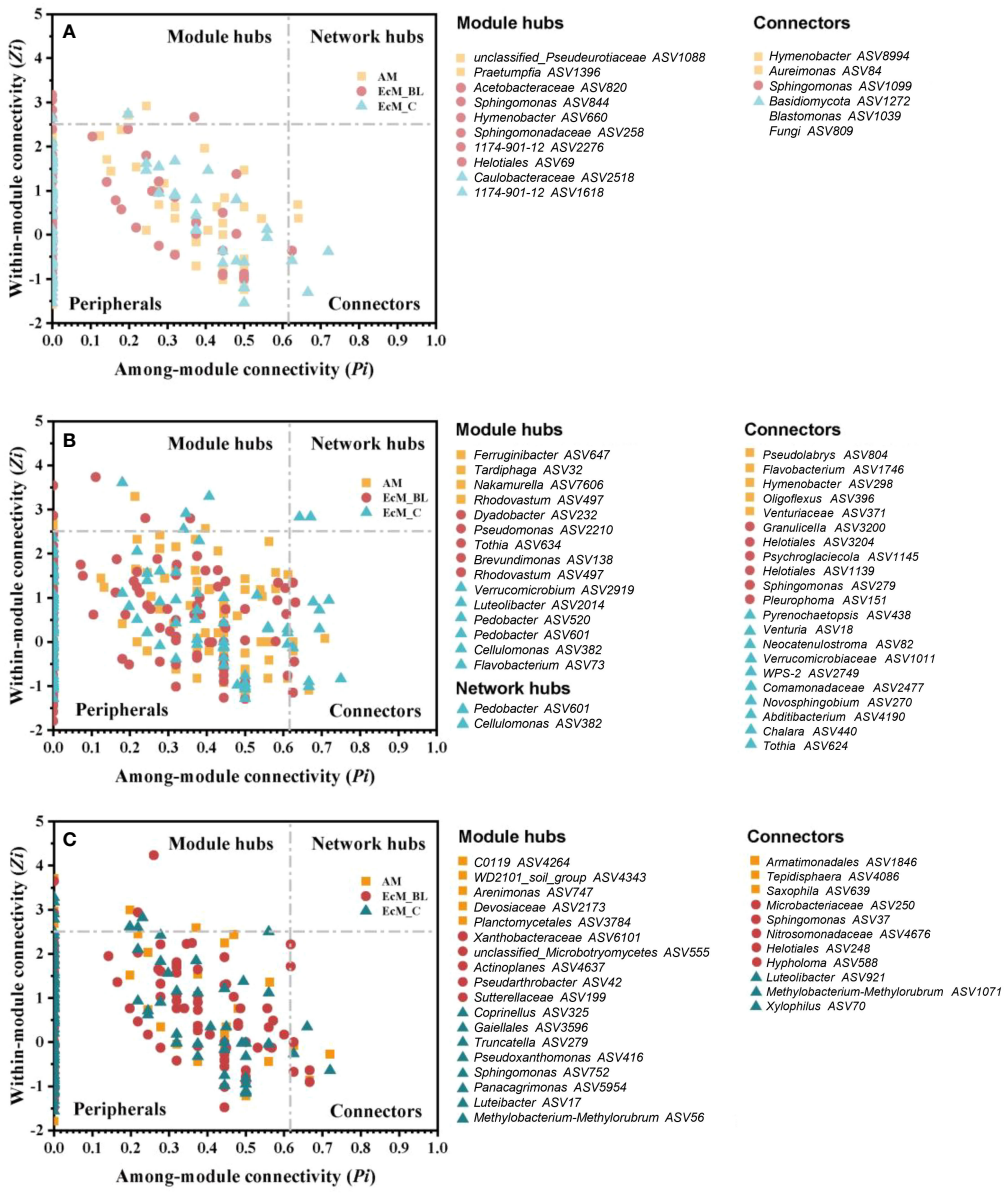
Topological roles of ASVs at 0 days **(A)**, 200 days **(B)**, and 400 days **(C)** decayed phase networks, as displayed by the *Zi*–*Pi* plot. AM_BL, broadleaved arbuscular mycorrhizal trees; EcM_BL, broadleaved ectomycorrhizal trees; EcM_C, coniferous ectomycorrhizal trees. The connectivity of each node was calculated based on its within-module connectivity (*Zi*) and among-module connectivity (*Pi*) to identify the keystone species according to Olesen et al., 2007.

The co-occurrence networks between bacteria and fungi (interkingdom) varied between the mycorrhizal types and sampling times (**Figure 4**). The divergent network topologies showed an obvious shift between broadleaved (AM_BL and EcM_BL) and coniferous (EcM_C) trees (**Figures 4A–C**). Notably, the network complexity of the interkingdom network was higher in EcM_BL trees than in the AM_BL and EcM_C groups (**Table S7**). Compared with conifers, the litter-associated interkingdom at 0 days and 200 days in AM_BL and EcM_BL trees had more nodes and a higher average degree (**Table S7**). The lower percentage of negative links in EcM_C trees indicated that the interkingdom network in conifers was more connected among ASVs and that there was less competition among ASVs than in broadleaved trees (**Table S7**). Interestingly, the total number of links and the proportion of negative links from all mycorrhizal types increased at 200 days and then decreased at 400 days, indicating more competition at a later stage of litter decomposition in forests (**Table S7**). N-fixing bacteria (*Brevundimonas*, *Methylobacterium-Methylorubrum*, *Pseudomonas*, and *Sphingomonas*), saprotrophic fungi (*Chalara*, *Coprinellus*, *Hypholoma*, *Praetumpfia*, and *Tothia*), and plant pathogenic fungi (*Neocatenulostroma*, *Pleurophoma*, *Truncatella*, and *Venturia*) were identified as network module hubs and connectors (**Figure 5**). *Sphingomonas* was identified as network connectors and/or module hubs in co-occurrence networks across sampling times.

### Factors influencing the leaf and needle microbial communities

Overall, we found that the tree mycorrhizal types influenced most of the leaf physicochemical properties (*P* < 0.001, **Table S6**). We found that plot factors and leaf physicochemical properties were also significantly correlated with the microbial community composition (both bacteria and fungi, **Table 1**). The plot factors and leaf physicochemical properties showed significant responses to microbial community composition across all sampling times included soil water content (*R* ^2^ = 0.23–0.65, *P* < 0.001), latitude (*R* ^2^ = 0.42–0.70, *P* < 0.001), leaf water content, pH, total C, N:P ratio, Ca, Fe, and Mg concentration (*R* ^2^ = 0.15–0.72, *P* < 0.05–0.001). Latitude was also the main factor corresponding to the fungal community composition (*R* ^2 = ^0.70, *P* < 0.001, **Table 1**), while total C content was another main factor for bacterial community composition at 0 day (*R* ^2^ = 0.72, *P* < 0.001, **Table 1**). Notably, DOC and N content (total N, dissolved inorganic N, and organic N) were significantly correlated with bacterial and fungal communities only at 0 days (*R* ^2^ = 0.16–0.62, *P* < 0.001–0.05).

When considering AM_BL, EcM_BL, and EcM_C separately, we found similar and dissimilar patterns of factors corresponding to bacterial and fungal community compositions (**Table S8**). Similar patterns that were observed across all considerations (AM_BL, EcM_BL, and EcM_C) were as follows: i) tree species was the main factor affecting the fungal community composition across all sampling times (*R* ^2^ = 0.83–1.00, *P* < 0.001), and ii) sampling times were the main factors corresponding to both bacterial and fungal community compositions (*R* ^2^ = 0.79–0.90, *P* < 0.001). The leaf and soil pH patterns were the first to differ. Leaf pH correlated significantly with bacterial and fungal community composition at most sampling times (**Table S8**). While leaf pH continued to significantly correlate with microbial community composition in AM_BL trees at 200 and 400 days, soil pH became more important for the microbial community composition in EcM_BL and EcM_C at 200 and 400 days (**Table S8**). Second, total C and DOC showed no significant correlation with the microbial community composition of AM_BL trees at 0 days, but they were significantly correlated with the microbial community composition of EcM_BL trees and were the main factors determining the microbial community composition of EcM_C trees (**Table S8**). Third, C:P and N:P ratios were the main factors that significantly corresponded to the fungal community composition in EcM_C trees at 0 days and C:N, C:P, and N:P ratios at 400 days; however, in other tree mycorrhizal types, they showed only moderate correlation or no correlation with the microbial community composition (**Table S8**). Details of enzyme activities and their association with microbial communities are provided in the **Supplementary Material**.

## Discussion

### Microbial richness, community composition, and their corresponding factors in nine tree species representing different tree mycorrhizal types

The results of this study confirmed our hypothesis that the mycorrhizal type of the tree affects nutrient acquisition and, thus, the nutrient traits of mature leaves and needles, which in turn determine the microbial community composition in the mature leaves and needles (**Figure 1**; **Table 1**). The effect of mycorrhizal type on microbial community composition was high at time 0 (mature leaves and needles) and decreased at later stages of decomposition. Previous studies (Bonfante and Genre, 2010; Jacobs et al., 2018; Keller and Phillips, 2019; Seyfried et al., 2021) have reported that tree mycorrhizal types play an important role in determining the nutrient traits of leaf litter and, thus, in selecting the phyllosphere microbiome prior to leaf senescence. Indeed, we found that the patterns of microbial community composition were correlated with nutrient content in different tree mycorrhizal types (**Table 1**). In mature leaves and needles before leaf senescence, DOC and N content (total N, dissolved inorganic N, and organic N) were more important in mature leaves and needles. Leachable C and N are the key components in the production of leaf litter-decomposing enzymes (Hoppe et al., 2014; Purahong et al., 2016). These two factors, namely, the initial phyllosphere microbiome and nutrient traits in the leaf litter, represent leaf litter quality and will later determine the leaf litter decomposition rate (Strickland et al., 2009). Indeed, we found that the leaf litter of AM trees decomposed faster than the leaves and needles of EcM broadleaves and conifers. This finding is consistent with a previous study of investigation on leaf litter decomposition in temperate forests (Keller and Phillips, 2019).

The succession of microbial community composition during leaf litter decomposition and its relationship to leaf litter quality is of great interest to scientists in various fields, especially ecology, microbiology, and forestry (Purahong et al., 2016; Tláskal et al., 2016). Nevertheless, previous studies (Purahong et al., 2016; Tláskal et al., 2016) have investigated microbial succession or only bacterial succession in one or two tree species. In this study, we revealed the microbial community dynamics and succession during leaf litter decomposition of nine temperate tree species, including broadleaf AM, broadleaf EcM, and conifer EcM. *Caulobacter*, *Flavobacterium*, *Brevundimonas*, *Rhizobiaceae*, *Polaromonas*, *Botryosphaeriales*, *Herpotrichia*, and *Chaetomium* were able to enrich the decomposing leaves and needles at 200 and 400 days, whereas *Sphingomonas*, *Massilia*, *Helotiales*, *Aureobasidium*, *Mycosphaerellaceae*, and *Didymellaceae* were drastically reduced at 200 and 400 days (**Figures S1–S4**). Interestingly, fungal enrichment was more specific to certain mycorrhizal tree types. Nevertheless, an increase in the relative abundance of a taxon can be explained by several different scenarios (Harrison et al., 2021). Thus, the absolute number of reads was checked to validate the above interpretation. The enrichment patterns of these microbes were mostly conserved when the absolute count of the sequence reads was considered. Nevertheless, further methods, such as the incorporation of internal standards of known quantity (Harrison et al., 2021) or quantitative PCR (Tanunchai et al., 2023), should be considered to better and directly approximate the absolute abundances in samples.

### More cross-kingdom competition in the early stages of different mycorrhizal litter decomposition

The relationship between bacteria and fungi (based on co-occurrence network patterns) differed among the tree mycorrhizal types during the decomposition period, which was consistent with our hypothesis. Network analysis can be used to determine how the environment affects an underlying network of ecological dependencies (Montoya et al., 2006). Given the characteristics of leaf habitats, cross-kingdom interactions in leaf microbial networks respond rapidly to environmental fluctuations (Barranca et al., 2015). Identifying bacterial and fungal associations within and between litter decomposition communities is critical for understanding this process (Purahong et al., 2016). In the present study, the leaf-associated microbial community members colonizing broadleaved trees were more sensitive to environmental variation than those colonizing conifers, and there was less cross-kingdom competition in the EcM_C group (**Figure 4**). Broadleaved trees tend to have more “opportunistic” strategies and longer leaf lives than evergreens (Reich, 1995; Wright et al., 2004). AM litter is more readily decomposed than EcM litter due to its higher nutrient and polyphenol content, which is conducive to the growth of fungal pathogens and saprotrophs that rely on C and energy from the plant litter (Averill et al., 2019; Keller and Phillips, 2019). Fang et al. (2020) demonstrated that a higher abundance of saprotrophic fungi in the soil around AM trees results in a faster decomposition rate for AM leaf litter compared with EcM leaf litter.

More importantly, the average clustering coefficient was relatively higher at 0 days than at 200 and 400 days (**Figure 4**; **Table S7**), suggesting that the bacterial–fungal network is less connected at later stages and may be more susceptible to external perturbations such as environmental influences. The theory of priority effects suggests that in the early stages of colonization, an early colonist can preempt niches and exclude later-arriving species by competing with them for resources (Cline and Zak, 2015). To the best of our knowledge, fungi have the potential to compete with bacteria for resources and expel them from their territories during the early decomposition phase (Tsujiyama and Minami, 2005; Purahong et al., 2016). In this study, we found that the percentage of links and negative links increased at 200 days and then decreased at 400 days, suggesting that more competitive and antagonistic relationships were present at the early stage of litter decomposition. We inferred that the competition potential of the cross-kingdom varied during the decomposition process due to niche differentiation. Cline and Zak (2015) showed that bacterial communities were decoupled from environmental changes and relatively stable despite different decomposition phases, mainly benefiting from readily available substances formed by fungal exoenzymes. Taken together, our network analyses (**Figure 4**) revealed that microbial interactions changed with the succession of litter decomposition and showed a contrasting pattern between mycorrhizal types.

### Variable selection and drift play important roles in regulating community assembly of leaf-associated microbiota

The proportion of determinisms and stochasticity that govern microbial communities change differently depending on the tree mycorrhizal type throughout the course of litter decomposition. Accumulating evidence suggests that plant-associated microbial communities are not random assemblages but rather are characterized by general rules for assembly and have well-defined phylogenetic relationships (Carlström et al., 2019). Assessing the ecological processes that govern the community assembly of leaf-associated microbiota provides a future avenue for advancing our understanding of litter decomposition (Carlström et al., 2019). In this study, the community assembly of leaf-associated bacteria was dominated by variable selection with a striking increase in the proportion of deterministic processes over time. This selective filtering and recruitment of different microorganisms can be attributed to niche adaptation and modification (Maignien et al., 2014; Hamonts et al., 2018). Several successful colonizers inhabiting litter niches either compete for resources or cooperate to maintain stable coexistence (Trivedi et al., 2020). Cline and Zak (2015) revealed that deviations in community assembly are significantly influenced by the respiration of early colonizers in the early stages. In addition, we found that stochastic processes (mainly drift) governed the assembly of fungal communities inhabiting the EcM leaves and needles (**Figure 3**). Vellend (2010) and Chase and Myers (2011) reported that drift is more prominent under conditions of reduced biodiversity (e.g., smaller populations and/or lower species richness). It has also been reported that a smaller habitat space and a lower dispersal rate, which are characteristic of needles, can increase stochasticity, especially ecological drift (Hanson et al., 2012; Evans et al., 2017). We observed an increasing trend in stochasticity in broadleaved trees (AM_BL and EcM_BL groups) during litter decomposition (**Figure 3**). Previous studies have suggested that initial plant colonization is favored by high-resource conditions, resulting in strong priority effects and divergent community assembly trajectories (Ejrnæs et al., 2006; Kardol et al., 2013). There is increasing evidence that the decomposition of N-rich, labile AM leaf litter results in increased mineral N availability and SOM content relative to EcM soil (Phillips et al., 2013; Keller and Phillips, 2019). Overall, community assemblages of leaf-associated microbiota are a multistep process that depends on species interactions, drift, and environmental selection.

### Link between enzyme activities and microbial communities

In this study, we found a correlation between enzyme activity and bacterial and fungal communities. Although fungi are known for their important role in decomposing complex biopolymers, bacteria can play direct and indirect roles in degrading complex leaf litter (de Gonzalo et al., 2016; Purahong et al., 2016). We found that the mass loss of different tree mycorrhizal types was directly related to the presence of some bacterial functional groups, especially N-fixing bacteria. We observed that the mass loss of needles from the EcM_C tree after 200 days of decomposition was significantly lower than that of the leaves from AM_BL and EcM_BL trees (**Figure 2**). Brown rot and ascomycetous fungi dominate the fungal community composition in mature (0 days) and decomposing leaves and needles at 200 days. We further found that at 0 days (mature leaves and needles), N-fixing bacteria, *Massilia*, *Methylobacterium*-*Methylorubrum*, *Pseudomonas*, and *Sphingomonas* were highly abundant in senescing leaves of AM_BL and EcM_BL trees, whereas only *Sphingomonas* was abundant in the senescing needles of the EcM_C tree (**Figure S2**). *Pseudomonas* dominated the bacterial community composition after 200 days of decomposition, whereas *Sphingomonas* co-dominated. *Pseudomonas* has been reported to be both an N-fixing bacterium and to produce a different type of peroxidase, bacterial DyP-type peroxidase, that modifies lignin (de Gonzalo et al., 2016; Desnoues et al., 2003). Saprotrophic fungi require available N to produce exoenzymes for leaf litter decomposition (Hoppe et al., 2014; Purahong et al., 2016). Thus, N availability may be the rate-limiting step in leaf litter decomposition (Osono and Takeda, 2005). The high mass loss at 400 days (up to 89%) was associated with an increase in enzyme activity, especially oxidative enzyme activity. This is consistent with the enrichment of *Mycena*, which has been reported to decompose lignin (Miyamoto et al., 2000; Kellner et al., 2014; Purahong et al., 2016). Furthermore, we found a link between oxidative enzyme activity and the bacterial community at 400 days (**Figure S5**). Bacteria may not directly secrete the measured oxidative enzymes; however, some bacteria (such as *Sphingobium* and *Novosphingobium*) have been reported to secrete glutathione-dependent enzymes (β-etherases and lyases) that act on the lignin degradation process (de Gonzalo et al., 2016). A full discussion of the relationship between enzyme activity and the microbial community is provided in the **Supplementary Material**.

## Conclusion

The fungal and bacterial community compositions of AM and EcM broadleaved trees as well as EcM conifer trees have only been studied for a few tree species. In this comprehensive study, we demonstrated for the first time that tree mycorrhizal types are critical for nutrient status, molar mass loss, and hydrolytic and oxidative enzyme activities of the microbial community and, thus, for the ecosystem service of leaf/needle decomposition. Moreover, each tree mycorrhizal type showed a specific community assembly process, and the microbial network architecture of broadleaved AM and EcM trees was more similar to each other than that of conifer EcM trees. Future research should use manipulation experiments to investigate the interactions of the community members, which were very abundant in our study, to decipher their respective roles in the leaf or needle decomposition process.

## Data availability statement

Illumina sequencing of bacterial and fungal datasets for 0, 200, and 400 days presented in the study are deposited in The National Center for Biotechnology Information (NCBI) database under BioProject ID PRJNA753096 and PRJNA890590.

## Author contributions

WP and E-DS conceived of and designed the study. BT, WP, SW, and E-DS led the experimental setup. BT, LJ, WP, SW, and E-DS collected the samples and metadata. WP, MN, and FB contributed the reagents and laboratory equipment. BT, KT, and WP performed the DNA analysis. SW led the bioinformatics analysis. BT, LJ, and WP led the microbial taxonomy and data analyses. LJ led the microbial network and community assembly analyses. SS, IH, and GG led the physicochemical analyses. BT, LJ, and WP drafted the manuscript. MN and WP supervised BT. MN, E-DS, and FB reviewed the manuscript and provided comments and suggestions. All authors contributed to the article and approved the submitted version.
