# Supplementary material for: Tree mycorrhizal type regulates leaf and needle microbial communities, affects microbial assembly and co-occurrence network patterns, and influences litter decomposition rates in temperate forest
Source: Front Plant Sci. 2023 Nov 29;14:1239600. doi: 10.3389/fpls.2023.1239600 (PMC10716483; doi:10.3389/fpls.2023.1239600)

Supplementary Material

Tree mycorrhizal type regulates leaf and needle microbial communities, affects microbial assembly and co-occurrence network patterns, and influences litter decomposition rates in temperate forest.

Benjawan Tanunchai[1,2,3¶], Li Ji[1,4¶], Simon Andreas Schroeter[5], Sara Fareed Mohamed Wahdan[1,6], Katikarn Thongsuk[1], Ines Hilke[5], Gerd Gleixner[5], François Buscot[1,7], Ernst-Detlef Schulze[5], Matthias Noll[2,3*], Witoon Purahong[1*]

[1]Department of Soil Ecology, UFZ-Helmholtz Centre for Environmental Research, Theodor-Lieser-Str. 4, 06120 Halle (Saale), Germany.

[2]Institute of Bioanalysis, Coburg University of Applied Sciences and Arts, Coburg, Germany.

[3]Bayreuth Center of Ecology and Environmental Research (BayCEER), University of Bayreuth, Bayreuth, Germany.

[4]School of Forestry, Central South of Forestry and Technology, 410004, Changsha, P.R. China.

[5]Max Planck Institute for Biogeochemistry, Biogeochemical Processes Department, Hans-Knöll-Str. 10, 07745 Jena, Germany.

[6]Department of Botany and Microbiology, Faculty of Science, Suez Canal University, 41522 Ismailia, Egypt.

[7]German Centre for Integrative Biodiversity Research (iDiv), Halle-Jena-Leipzig, Deutscher Platz 5e, 04103 Leipzig, Germany.

***Correspondence**: benjawan.tanunchai@hs-coburg.de; witoon.purahong@ufz.de; witoon.purahong@gmail.com; Matthias.noll@hs-coburg.de; Tel.: 09561-317645

¶ These authors contributed equally to this work

Material and methods

Study site, experimental setup, designs

Additional details of the study site are provided in this Supplementary Material. Briefly, the characteristics of this site are elevations from 100 to 494 m.a.s.l., average annual precipitation from 600 to 800 mm, average annual temperatures from 6 to 7.5 °C, average temperature in January = 0.65°C and July = 17.17°C. The main soil type is Cambisol on limestone as bed rock with an average soil pH of 5.1 ± 1.1; mean ± SD. Most sites are covered by a litter layer consisting mainly of past years foliage (1 to 3 cm), followed by a Pleistocene loess layer of variable thickness (ca. 10–50 cm), underlain by Triassic limestone parent material. All the analyses are summarized in the experimental scheme.

Experimental scheme

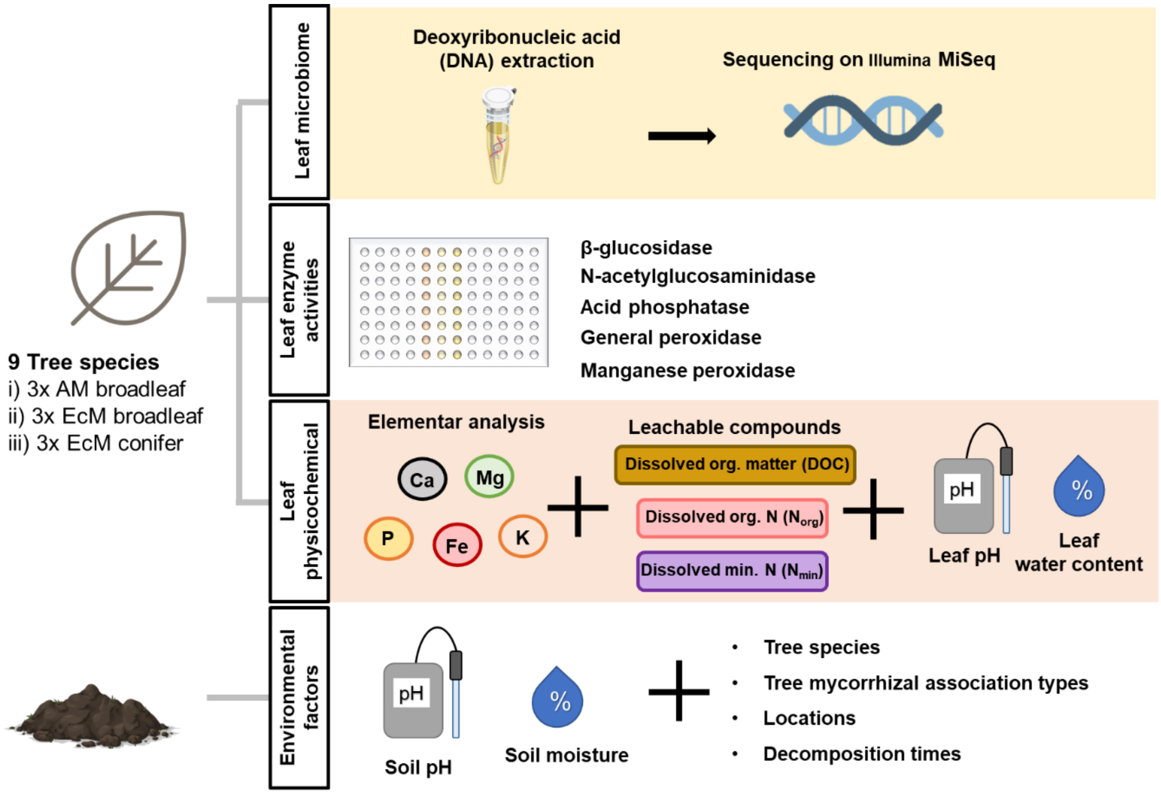

Physiochemical analyses

The procedures for the physiochemical analyses were published by Tanunchai et al. (2022a) and are presented in the Supplementary Material. Briefly, leachable components were extracted by incubating wet leaf and needle samples in 30 mL MilliQ water for 1 h at room temperature, centrifuging for 5 min at 2889 × g, decanting, and filtering through pre-flushed 0.45 µm regenerated cellulose syringe filters. The dry weight, pH, and water content of the leaves and needles were measured after two weeks of oven drying at 40 °C. All quantification results are presented in reference to the dry weight. Total leaf C (C), total leaf N (N), nutrient (including Ca, Fe, K, Mg, and P content), dissolved organic C (DOC), dissolved organic N (N_{org}), and dissolved inorganic N (N_{min}) contents were analyzed. N_{Min}, ammonium-N, nitrite-N, and nitrate-N, including nitrite-N, were quantified using a flow injection analyzer (Quikchem QC85S5; Lachat Instruments, Hach Company, Loveland CO, USA). DOC was measured using a sum parameter analyzer with high-temperature combustion and infrared detection (Vario TOC cube, Elementar Analysensysteme GmbH, Langenselbold, Germany). Nutrient ions and Ca, Fe, K, Mg, and P contents were quantified using inductively coupled plasma optical emission spectrometry (ICP–OES, PerkinElmer Inc., Waltham, MA, USA). More details on the physicochemical analyses have been published by Tanunchai et al., 2022a.

Enzyme analyses

Five potential enzyme activities, including three hydrolytic enzymes (β-glucosidase, N-acetylglucosaminidase, and acid phosphatase) and two oxidative enzymes, including peroxidase and manganese peroxidase (Purahong et al., 2016b), were measured from homogenized leaf litter samples (0.1 g wet weight). Hydrolytic and oxidative enzyme activities were measured using a fluorometric microplate assay (TECAN Infinite F200Pro, Grödig, Austria) with 4-

methylumbelliferone (MUF) (Sigma-Aldrich, Steinheim, Germany) and 3,3′,5,5′-tetramethylbenzidine (TMB) (Sigma-Aldrich)-labeled substrates, respectively, as described previously (Purahong et al., 2016a). The leaf samples were weighed in wet weight, but all enzyme activity results were later calculated and given in reference to the leaf dry weight.

DNA extraction, Illumina sequencing, and bioinformatics

Mature leaves and needles (0 day), healthy leaves (up to 10 leaves per tree individual, depending on leaf size), and needles (from five branches per tree individual) were subsampled and prepared for DNA extraction. For decomposing leaves and needles at 200 and 400 days of incubation, 1 g of leaves and needles were subsampled. The leaf and needle samples were washed and DNA was extracted using the DNeasy PowerSoil Kit (Qiagen, Hilden, Germany). The presence and amount of DNA were checked using a NanoDrop ND-1000 spectrophotometer (Thermo Fisher Scientific, Dreieich, Germany), and the extracts were stored at −20 °C.

For the library preparation, the polymerase chain reactions (PCR) were performed using 20 µL reaction volumes with 5× HOT FIRE Pol Blend Master Mix (Solis BioDyne, Tartu, Estonia) on ABI Veriti thermocyclers (Applied Biosystems, Carlsbad, CA, USA). The bacterial polymerase chain reaction (PCR) conditions were as follows:95 °C for 15 min, followed by 30 cycles of 95 °C for 20 s, 56 °C for 30 s, 72 °C for 1 min, followed by one extension cycle at 72 °C for 5 min, and a 4 °C hold. The fungal PCR conditions were 95 °C for 15 min, followed by 30 cycles of 95 °C for 20 s, 54 °C for 40 s, and 72 °C for 1 min, followed by one extension cycle at 72 °C for 10 min, and a 4 °C hold. The amplified products were visualized using gel electrophoresis. Amplification products (three replicate reactions per sample) were pooled in equimolar amounts and purified using an Agencourt AMPure XP kit (Beckman Coulter, Krefeld, Germany). Illumina Nextera XT Indices were added at both ends of the fungal amplicons. Paired-end sequencing (2 × 300 bp) was

performed at the Department of Soil Ecology, Helmholtz Centre for Environmental Research, Germany.

Bioinformatics

Assembled reads fulfilling the following criteria were retained for further analyses: a minimum length of bacterial forward sequences of 200 nucleotides (nt) and bacterial reverse sequences of 150 nt, a minimum length of fungal forward and reverse sequences of 70 nt, quality scores at least equal to 15 (bacteria) and 9 (fungi), with a maximum expected error score of 5 for forward and reverse sequences, and no ambiguous nucleotides. Merging was conducted with two mismatches allowed, and a minimum overlap of 20 nucleotides was required for bacterial and fungal sequences. High-quality reads were clustered into amplicon sequence variants (ASVs). The SILVA SSU database v. 138 was used for taxonomic classification of bacterial ASVs (Yilmaz et al., 2014). Fungal ASVs were classified against the UNITE v7.2 database (Kõljalg et al., 2013). The set of ASVs was classified using the Bayesian classifier, as implemented in the mothur classify.seqs command, with a cut-off of 60. Rare ASVs (singletons) were excluded. The datasets were then rarefied to the minimum sequencing depth of the bacterial and fungal sequence reads. The rarefaction curves of all samples reached saturation (Figure provided below). The Mantel test based on the Bray–Curtis distance measure with 999 permutations was applied to evaluate the correlation between the whole matrix and a rarified matrix for the bacterial and fungal datasets (Tanunchai et al., 2022b).

Rarefaction curves of both bacterial and fungal datasets.

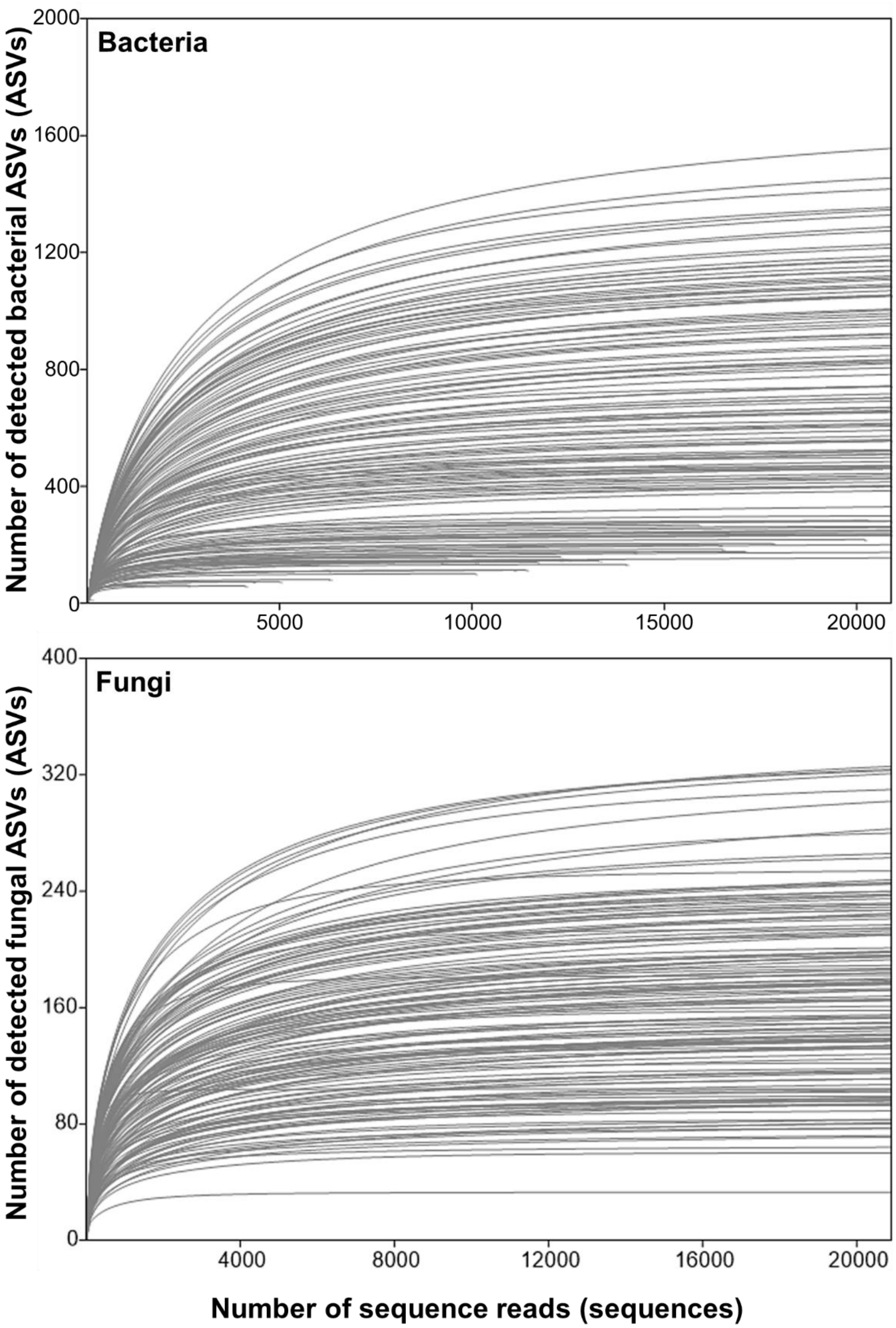

Network and community assembly analyses

The connectivity of each node was calculated based on its within-module connectivity (*Zi*) and among-module connectivity (*Pi*) to identify the keystone species according to Olesen et al., 2007. Four criteria of node topologies were identified: (1) module hubs (highly connected nodes within modules, *Zi* > 2.5); (2) network hubs (highly connected nodes within the entire network, *Zi* > 2.5 and Pi > 0.62); (3) connectors (nodes connecting modules, *Pi* > 0.62), and (4) peripherals (interconnected nodes in modules with few outside connections, *Zi* < 2.5 and *Pi* < 0.62). All networks were visualized using Gephi v0.9.2. All above mentioned parameters were calculated by 'iCAMP' package in R with the code provided by (Ning et al., 2020) (https://github.com/DaliangNing/iCAMP1).

Results

General overview of the leaf and needle microbiome in forest ecosystems

Mature (0 day) and decomposing (early stage 200 and late stage 400 days) leaves and needles of nine temperate tree species were colonized by 14,773 bacterial and 4,884 fungal ASVs (belonging to 585 bacterial and 594 fungal genera). The bacterial classes; Alphaproteobacteria (30.5 – 42.7% relative sequence read abundance, represented by *Sphingomonas*, uncultivated *Beijerinckiaceae* [1174-901-12], and *Brevundimonas*), Grammaproteobacteria (21.5 – 31.7% relative sequence read abundance, represented by *Massilia*, *Pseudomonas*, and *Variovorax*), Bacteroidia (19.3 – 23.1% relative sequence read abundance, represented by *Hymenobacter*, *Pedobacter*, and *Flavobacterium*), and Actinobacteria (6.9 – 11.1% relative sequence read abundance, represented by *Microbacteriaceae*) formed the backbone in mature and decomposing leaves and needles of all tree mycorrhizal species and decomposition stages (Fig. S1 and Table S2). Nevertheless, the patterns of these backbone bacterial classes differentiated in broadleaved arbuscular (AM_BL),

broadleaved ectomycorrhizal (EcM_BL), and coniferous ectomycorrhizal (EcM_C) trees, and shifted during leaf litter decomposition (Fig. S1). Remarkably, N-fixing bacteria (*Sphingomonas*, *Pseudomonas*, *Allorhizobium-Neorhizobium-Pararhizobium-Rhizobium*, and *Methylobacterium-Methylorubrum*) dominated the bacterial community composition across the tree mycorrhizal types over the decomposition time (Fig. S1). Aerobic Chemoheterotrophy was the most detected bacterial function (up to 85% relative sequence read abundance) across tree mycorrhizal types and decomposition time (Figure below).

The fungal community composition in mature and decomposing leaves and needles of all tree mycorrhizal types was dominated by the fungal phylum Ascomycota. Dothideomycetes (24.1 – 41.1% relative sequence read abundance, represented by *Alternaria*, *Aureobasidium*, and *Cladosporium*) and Leotiomycetes (26.6 – 42.0% relative sequence read abundance, represented by *Hymenoscyphus*, *Tetracladium*, and *Mollisina*), followed by Sordariomycetes (8.6 – 24.5% relative sequence read abundance, represented by *Chaetomium* and *Fusidium*) dominated the fungal community composition in leaves and needles of AM_BL, EcM_BL, and EcM_C trees across the decomposition time. The fungal class Tremellomycetes (9.4 % relative abundance, represented by *Vishniacozyma*) in the phylum Basidiomycota substantially contributed to the fungal community composition in mature leaves (0 day) of AM_BL and EcM_BL trees, which was later replaced by the fungal class Agaricomycetes (6.9 – 8.5% relative sequence read abundance, represented by *Mycena*) in the early (200 days) and late (400 days) stages of leaf litter decomposition. The pattern of fungal community composition in the needles of EcM_C trees differed from that in the leaves of AM_BL and EcM_BL trees. In the mature needles (0 day) of EcM_C trees, fungal classes in the fungal phyla Ascomycota and Eurotiomycetes (18.2% relative sequence read abundance, represented by *Chaetothyriales*) and Dothideomycetes (37.4% relative

sequence read abundance, represented by *Cladosporium* and *Phoma*) dominated the fungal community composition (Fig. S3 and Table S3). The relative sequence read abundance of Dothideomycetes increased with decomposition time (60.0% and 63.0% relative sequence read abundance at 200 days and at 400 days, respectively) and Eurotiomycetes was replaced by members of the fungal class Agaricomycetes (9.4% and 13.3% relative sequence read abundance at 200 and 400 days, represented by *Mycena*) in the phylum Basidiomycota. Saprotrophic and plant pathogenic fungi were dominant across all comparisons, regardless of tree mycorrhizal type and decomposition time (Figure below).

Relative proportions of functional groups of bacteria (a – c) and fungi (d – f). Subfigures show the functional groups of bacteria and fungi in different tree species (a, d), tree mycorrhizal types (b, e), and sampling times (c, f). Colored circles behind functional group names indicate significant differences.

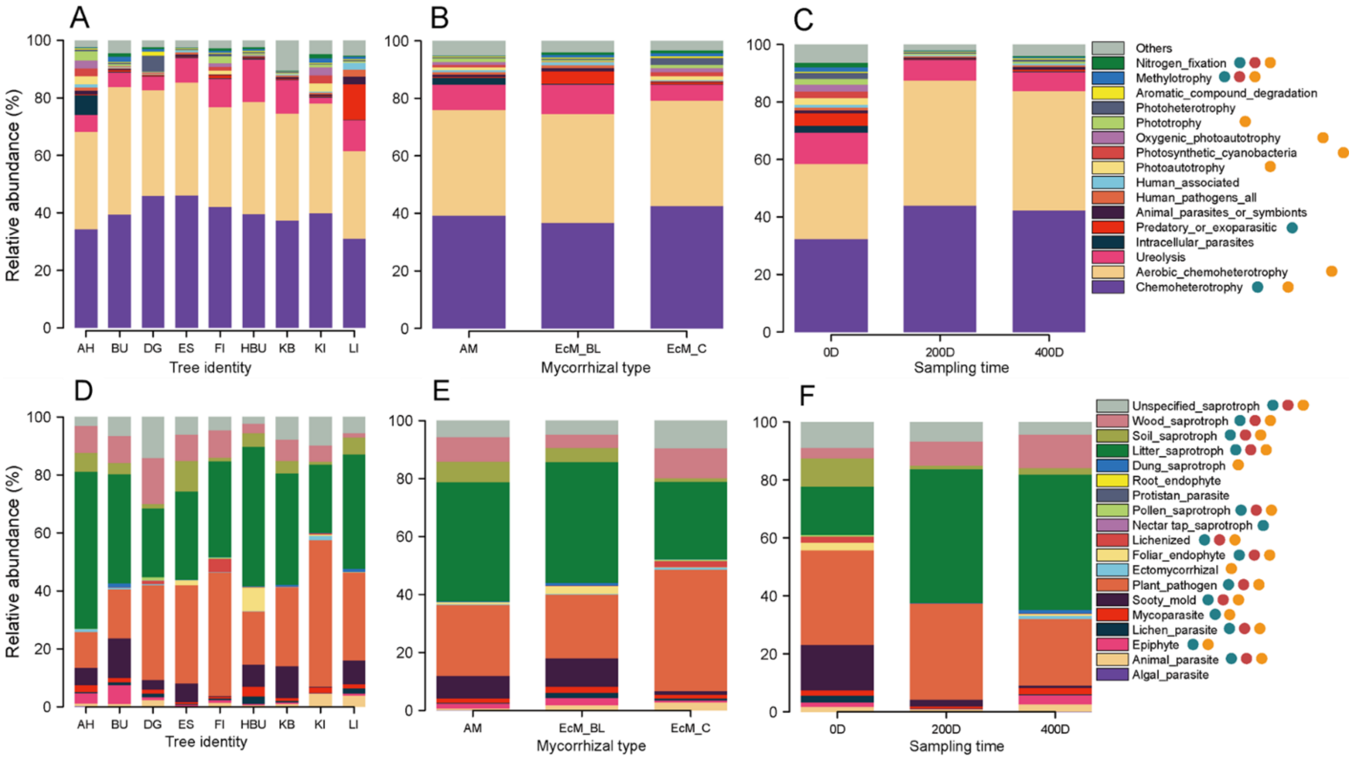

Enzyme activities and their links to microbial communities

Hydrolytic enzyme activity was significantly higher in the leaves of AM_BL and EcM_BL trees than in the needles of EcM_C trees (Fig. S5). The oxidative enzyme activities in the leaves and needles of different tree mycorrhizal types were low or almost absent after 200 days of decomposition (Fig. S5). The activities of both hydrolytic and oxidative enzymes significantly increased across tree mycorrhizal types at 400 days of decomposition (Fig. S5).

Overall, the bacterial and fungal community compositions were correlated with both hydrolytic and oxidative enzyme activities (Table S9). At 200 days of decomposition, the bacterial community composition in the leaves of the AM_BL and EcM_BL trees was correlated with hydrolytic enzyme activities (R^2 = 0.55 – 0.93, P < 0.05 – 0.001, Table S9). No significant correlation between bacterial community composition and enzyme activities was observed in the needles of EcM_C trees at 200 days (Table S9). At 400 days of decomposition, the bacterial community composition in leaves and needles across all tree mycorrhizal types was moderately to highly correlated with both hydrolytic and oxidative enzyme activities (R^2 = 0.60 – 0.90, P < 0.01 – 0.001, Table S9). While the correlation between bacterial community composition and enzyme activities showed different patterns across decomposition times, the correlations between fungal community composition were rather consistent, especially when considering EcM_BL and EcM_C trees (Table S9).

Discussion

Link between enzyme activities and microbial communities

Further discussion of the relationship between enzyme activity and the microbial community is discussed here. Brown rot and ascomycetous fungi dominated the fungal community composition in mature (0 day) and decomposing leaves and needles at 200 days. The leaves of AM_BL and

EcM_BL trees were dominated by *Aureobasidium* in the fungal community composition, which was later replaced by *Alternaria*, *Chaetomium*, and Leotiomycetes groups, *Hymenoscyphus*, *Mollisina*, and *Tetracladium* after 200 days of decomposition (Fig. S3). Members of the Leotiomycetes group are specialists in decomposing biopolymers (such as cellulose, hemicellulose, pectin, and chitin) and produce cellulase enzymes (Schneider et al., 2012). Needles of EcM_C trees were mainly colonized by different groups of ascomycetous fungi (*Cladosporium*, *Herpotrichia*, and *Phoma*) after 200 days of decomposition (Fig. S3). White rot fungi, especially *Mycena*, become enriched after 200 days of decomposition. *Mycena* is a lignin decomposer that secretes laccase and manganese peroxidase (Miyamoto et al., 2000; Kellner et al., 2014; Purahong et al., 2016b). Although fungi are known for their important role in decomposing complex biopolymers, bacteria may play direct and indirect roles in leaf litter decomposition (de Gonzalo et al., 2016; Purahong et al., 2016b). We found that the mass loss of different tree mycorrhizal types was directly related to the presence of some bacterial functional groups, specifically N-fixing bacteria. We observed that the mass loss of needles of EcM_C trees after 200 days of decomposition was significantly lower than that of leaves from AM_BL and EcM_BL trees (Fig. 2). We found that on 0 day (mature leaves and needles), N-fixing bacteria, *Massilia*, *Methylobacterium-Methylorubrum*, *Pseudomonas*, and *Sphingomonas* were highly abundant in senescing leaves of AM_BL and EcM_BL trees, whereas *Sphingomonas* was abundant in the senescing needles of the EcM_C tree (Fig. S2). *Pseudomonas* dominated the bacterial community composition at 200 days of decomposition, while *Sphingomonas* co-dominated. Pseudomonas has been reported to be both an N-fixing bacterium and to produce another type of peroxidase, bacterial DyP-type peroxidases, that modify lignin (de Gonzalo et al., 2016; Desnoues et al., 2003). Saprotrophic fungi require available N to produce exoenzymes for leaf litter decomposition

(Hoppe et al., 2014; Purahong et al., 2016b). Thus, N availability may be the rate-limiting step in leaf litter decomposition (Osono and Takeda, 2005). The high mass loss at 400 days (up to 89%) was coupled with an increase in enzyme activity, especially oxidative enzyme activity. Although hydrolytic enzymes are important for the acquisition of polymeric C (β-glucosidase), N (N-acetylglucosaminidase), and P (acid phosphatase), the two oxidative enzymes (general peroxidase and manganese peroxidase) are responsible for the modification or breakdown of complex biopolymers such as lignin. (Purahong et al., 2016b). This is consistent with the enrichment of *Mycena*, which has been reported to decompose lignin (Miyamoto et al., 2000; Kellner et al., 2014; Purahong et al., 2016b). Furthermore, we found a link between oxidative enzyme activity and the bacterial community at 400 days (Fig. S5). Bacteria may not directly secrete the measured oxidative enzymes; however, some bacteria (such as *Sphingobium* and *Novosphingobium*) have been reported to secrete glutathione-dependent enzymes (β-etherases and lyases) that act on the lignin degradation process (de Gonzalo et al., 2016).

References

de Gonzalo, G., Colpa, D. I., Habib, M. H. M., and Fraaije, M. W. (2016). Bacterial enzymes involved in lignin degradation. *Journal of Biotechnology* 236, 110–119. doi: 10.1016/j.jbiotec.2016.08.011.

Desnoues, N., Lin, M., Guo, X., Ma, L., Carreño-Lopez, R., and Elmerich, C. (2003). Nitrogen fixation genetics and regulation in a *Pseudomonas stutzeri* strain associated with rice. *Microbiology* 149, 2251–2262. doi: 10.1099/mic.0.26270-0.

Hoppe, B., Kahl, T., Karasch, P., Wubet, T., Bauhus, J., Buscot, F., et al. (2014). Network analysis reveals ecological links between N-fixing bacteria and wood-decaying fungi. *PLoS ONE* 9, e88141. doi: 10.1371/journal.pone.0088141.

Kellner, H., Luis, P., Pecyna, M. J., Barbi, F., Kapturska, D., Krüger, D., et al. (2014). Widespread occurrence of expressed fungal secretory peroxidases in forest soils. *PLoS ONE* 9, e95557. doi: 10.1371/journal.pone.0095557.

Kõljalg, U., Nilsson, R. H., Abarenkov, K., Tedersoo, L., Taylor, A. F. S., Bahram, M., et al. (2013). Towards a unified paradigm for sequence-based identification of fungi. *Mol Ecol* 22, 5271–5277. doi: 10.1111/mec.12481.

Miyamoto, T., Igarashi, T., and Takahashi, K. (2000). Lignin-degrading ability of litter-decomposing basidiomycetes from *Picea* forests of Hokkaido. *Mycoscience* 41, 105–110. doi: 10.1007/BF02464317.

Ning, D., Yuan, M., Wu, L., Zhang, Y., Guo, X., Zhou, X., et al. (2020). A quantitative framework reveals ecological drivers of grassland microbial community assembly in response to warming. *Nat Commun* 11, 4717. doi: 10.1038/s41467-020-18560-z.

Olesen, J. M., Bascompte, J., Dupont, Y. L., and Jordano, P. (2007). The modularity of pollination networks. PNAS 104, 19891–19896. doi: 10.1073/pnas.0706375104.

Osono, T., and Takeda, H. (2005). Decomposition of organic chemical components in relation to nitrogen dynamics in leaf litter of 14 tree species in a cool temperate forest. *Ecological Research* 20, 41–49. doi: https://doi.org/10.1007/s11284-004-0002-0.

Purahong, W., Durka, W., Fischer, M., Dommert, S., Schöps, R., Buscot, F., et al. (2016a). Tree species, tree genotypes and tree genotypic diversity levels affect microbe-mediated soil ecosystem functions in a subtropical forest. *Scientific Reports* 6, 36672. doi: 10.1038/srep36672.

Purahong, W., Wubet, T., Lentendu, G., Schloter, M., Pecyna, M. J., Kapturska, D., et al. (2016b). Life in leaf litter: novel insights into community dynamics of bacteria and fungi during litter decomposition. *Mol Ecol* 25, 4059–4074. doi: 10.1111/mec.13739.

Schneider, T., Keiblinger, K. M., Schmid, E., Sterflinger-Gleixner, K., Ellersdorfer, G., Roschitzki, B., et al. (2012). Who is who in litter decomposition? Metaproteomics reveals major microbial players and their biogeochemical functions. *ISME J* 6, 1749–1762. doi: 10.1038/ismej.2012.11.

Tanunchai, B., Ji, L., Schroeter, S. A., Wahdan, S. F. M., Hossen, S., Delelegn, Y., et al. (2022a). FungalTraits vs. FUNGuild: comparison of ecological functional assignments of leaf- and needle-associated fungi across 12 temperate tree species. *Microb Ecol*. 85, 411-428 doi: 10.1007/s00248-022-01973-2.

Tanunchai, B., Ji, L., Schroeter, S. A., Wahdan, S. F. M., Larpkern, P., Lehnert, A.-S., et al. (2022b). A poisoned apple: First insights into community assembly and networks of the fungal pathobiome of healthy-looking senescing leaves of temperate trees in mixed forest ecosystem. *Front. Plant Sci.* 13. doi: https://doi.org/10.3389/fpls.2022.968218.

Yilmaz, P., Parfrey, L. W., Yarza, P., Gerken, J., Pruesse, E., Quast, C., et al. (2014). The SILVA and "All-species Living Tree Project (LTP)" taxonomic frameworks. *Nucleic Acids Res* 42, D643–D648. doi: 10.1093/nar/gkt1209.

Table S1 Bacterial richness at different rarefaction depths. The abbreviations are AH: *Acer pseudoplatanus*, BU: *Fagus sylvatica*, DG: *Pseudotsuga menziesii*, ES: *Fraxinus excelsior*, FI: *Picea abies*, HBU: *Carpinus betulus*, KI: *Pinus sylvestris*, KB: *Prunus avium*, and LI: *Tilia cordata*.

Tree species	Sequence reads	Rarification depths							
		20000	15000	10000	6000	4000	2500	2000	1000
FI4	20787	459	456	446	430	406	365	350	267
FI3	20676	397	394	384	371	354	327	320	244
KI1	20479	283	282	277	265	258	239	229	179
BU1	20309	218	216	212	205	197	181	174	144
AH3	17946	205	205	200	195	188	168	167	137
ES4	17287	177	177	173	163	156	147	135	121
DG3	16602	185	183	182	174	167	147	149	131
HBU2	16550	199	198	193	183	176	161	153	131
KI5	16051	268	268	265	254	248	233	224	190
HBU1	15672	203	203	200	192	179	164	154	129
HBU4	14329	175	175	172	165	153	145	143	116
ES2	14187	133	133	132	129	122	117	114	94
DG5	13413	145	145	144	144	144	139	139	124
AH5	12693	148	148	146	144	136	135	120	113
DG4	12499	147	147	146	147	142	140	130	115
KI3	12436	159	159	157	155	150	143	140	123
LI5	11861	141	141	141	134	132	123	120	101
LI3	11660	146	146	144	140	131	128	126	113
KB2	11521	114	114	113	110	108	105	92	89
ES1	11208	113	113	113	109	110	97	99	86
LI4	10249	101	101	101	99	97	96	91	85
AH4	10204	140	140	140	136	131	130	119	102
KI2	10145	164	164	164	161	157	144	140	116
AH2	9493	156	156	156	152	147	136	135	114
KI4	9335	141	141	141	139	138	132	132	112
ES3	6426	81	81	81	80	78	74	75	65
LI1	6402	110	110	110	109	109	104	99	95
FI4.400	5160	186	186	186	186	186	180	177	155
KB5	5129	74	74	74	74	72	69	71	64
KB1	4479	74	74	74	74	74	72	71	66
ES5	4274	59	59	59	59	59	59	56	55
KB3	2733	62	62	62	62	62	62	62	58

Table S2 Information on the relative abundance (%) of bacterial ASVs detected in mature (0 day) and decomposing (200 and 400 days) leaves and needles in forest ecosystems (see accompanying Excel file).

Table S3 Information on the relative abundance (%) of fungal ASVs detected in mature (0 day) and decomposing (200 and 400 days) leaves and needles in forest ecosystems (see accompanying Excel file).

Table S4. Absolute number of reads of some bacteria and fungi.

Table S5 Comparisons between bacterial (a) and fungal (b) community compositions associated with mature (0 day) and decomposing (200 and 400 days) leaves and needles in forest ecosystems using analysis of similarities (ANOSIM) and non-parametric multivariate analysis of variance (NPMANOVA) based on relative abundance data and the Bray-Curtis distance measure (see accompanying Excel file).

Table S6. Effects of time and forest mycorrhizal type on leaf mass loss, microbial ASV richness, and physicochemical properties. Statistical differences were tested using repeated measures analysis of variance (ANOVA) with Fisher's Least Significant Difference (LSD) post-hoc test. Bold letters indicate statistical significance.

	Tree mycorrhizal type		
	Time	Mycorrhizal type	Time x mycorrhizal type
Leaf mass loss	F = 195.68<br>**P < 0.001**	F = 15.58<br>**P < 0.001**	F = 7.45<br>**P = 0.002**
Bacterial richness	F = 568.93<br>**P < 0.001**	F = 9.26<br>**P < 0.001**	F = 12.54<br>**P < 0.001**
Fungal richness	F = 1.70<br>P = 0.200	F = 5.20<br>**P = 0.010**	F = 35.67<br>**P < 0.001**
Leaf water content	F = 58.99<br>**P < 0.001**	F = 32.25<br>**P < 0.001**	F = 3.26<br>**P = 0.048**
Leaf pH	F = 36.31<br>**P < 0.001**	F = 15.21<br>**P < 0.001**	F = 3.62<br>**P = 0.035**
C	F = 77.82<br>**P < 0.001**	F = 47.45<br>**P < 0.001**	F = 6.58<br>**P = 0.003**
DOC	F = 130.05<br>**P < 0.001**	F = 21.71<br>**P < 0.001**	F = 30.48<br>**P < 0.001**
N	F = 139.97<br>**P < 0.001**	F = 1.52<br>P = 0.232	F = 0.79<br>P = 0.460
N_{min}	F = 87.64<br>**P < 0.001**	F = 8.70<br>**P < 0.001**	F = 4.34<br>**P = 0.020**
N_{org}	F = 24.94<br>**P < 0.001**	F = 7.93<br>**P = 0.001**	F = 24.53<br>**P < 0.001**
C: N ratio	F = 148.05<br>**P < 0.001**	F = 4.01<br>**P = 0.025**	F = 0.52<br>P = 0.596
C: P ratio	F = 2.52<br>P = 0.120	F = 17.41<br>**P < 0.001**	F = 1.89<br>P = 0.163
N: P ratio	F = 79.83<br>**P < 0.001**	F = 31.07<br>**P < 0.001**	F = 3.93<br>**P = 0.027**
Ca	F = 15.65<br>**P < 0.001**	F = 26.60<br>**P < 0.001**	F = 0.10<br>P = 0.910
Fe	F = 194.26<br>**P < 0.001**	F = 55.97<br>**P < 0.001**	F = 2.17<br>**P = 0.127**
K	F = 6.53<br>**P = 0.014**	F = 1.23<br>P = 0.304	F = 5.94<br>**P = 0.005**
Mg	F = 0.31<br>P = 0.581	F = 16.11<br>**P < 0.001**	F = 6.19<br>**P = 0.004**
P	F = 12.60<br>**P < 0.001**	F = 18.15<br>**P < 0.001**	F = 9.26<br>**P < 0.001**

Table S7 Topological properties of modular networks of leaf/needle-associated microbes.

Network features		0 day			200 days			400 days		
		AM	EcM_BL	EcM_C	AM	EcM_BL	EcM_C	AM	EcM_BL	EcM_C
Empirical network	Number of nodes	167	240	210	407	436	309	467	603	468
	Number of links	294	410	275	665	1020	463	488	796	613
	R^2 of power-law	0.908	0.906	0.778	0.907	0.865	0.940	0.920	0.910	0.910
	Number of positive correlations	206 (70.1 %)	277 (67.6 %)	208 (75.6 %)	523 (78.6 %)	855 (83.8 %)	354 (76.5 %)	340 (69.7 %)	635 (79.8 %)	425 (69.3 %)
	Number of negative correlations	88 (29.9 %)	1338 (32.4 %)	67 (24.4 %)	142 (21.4 %)	165 (16.2 %)	109 (23.5 %)	148 (30.3 %)	161 (20.2 %)	188 (30.7 %)
	Average degree (avgK)	3.521	3.417	2.619	3.268	4.679	2.997	2.090	2.640	2.620
	Average clustering coefficient (avgCC)	0.178	0.196	0.185	0.113	0.195	0.106	0.048	0.101	0.147
	Average path distance (GD)	5.553	7.106	8.34	6.284	6.018	5.818	7.312	7.472	6.892
	Modularity	0.704	0.690	0.817	0.669	0.621	0.727	0.854	0.808	0.843
Random network	avgCC±SD	0.030±0.010	0.026±0.007	0.010±0.006	0.016±0.004	0.043±0.006	0.013±0.004	0.003±0.001	0.006±0.001	0.007±0.003
	GD±SD	3.846±0.074	3.846±0.074	5.347±0.108	4.321±0.061	3.669±0.039	4.589±0.085	6.319±0.191	5.385±0.082	5.405±0.102
	Modularity±SD	0.518±0.009	0.518±0.009	0.669±0.010	0.572±0.006	0.426±0.004	0.610±0.007	0.800±0.007	0.689±0.006	0.690±0.007

Note: AM: arbuscular mycorrhizal. EcM_BL, broadleaf tree species belonging to the ectomycorrhizal association; EcM_C, coniferous tree species belonging to the ectomycorrhizal association.

Table S8 Goodness-of-fit statistics (R^2) of environmental variables fitted to non-metric multidimensional scaling (NMDS) ordination of bacterial and fungal communities in (a) AM_BL tree species, (b) EcM_BL, and (c) EcM_C based on relative abundance data and the Bray-Curtis distance measure. Bold letters indicate statistical significance with $R^2 \geq 0.70$. * P<0.05, ** P<0.01, *** P<0.001, NA = not applicable.

(a) AM_BL

**Factors**	**All sampling times**		**0 day**		**200 days**		**400 days**	
	**Bac**	**Fun**	**Bac**	**Fun**	**Bac**	**Fun**	**Bac**	**Fun**
**Tree factors**								
Tree mycorrhizal type	NA	NA	NA	NA	NA	NA	NA	NA
Tree species	0.05	0.32***	0.55**	**0.89*** **	0.60**	**0.84*** **	**0.77*** **	**0.87*** **
**Time factor**								
Sampling times	**0.90*** **	**0.87*** **	NA	NA	NA	NA	NA	NA
**Plot factors**								
Soil water content	0.05	0.33***	0.10	0.41*	0.09	0.22	0.24	0.43*
Soil pH	0.12	0.00	0.48*	**0.81*** **	0.18	0.30	0.23	0.39
Latitude	0.01	0.09	0.02	0.02	0.18	0.24	0.09	0.13
Longitude	0.25**	0.12	0.19	0.15	0.42*	0.35	0.31	0.13
**Leaf physicochemical properties**								
Leaf water content	0.19*	0.18*	0.10	0.43*	0.29	0.29	0.06	0.09
Leaf pH	0.17*	0.16*	0.58**	**0.90*** **	0.49*	0.54*	**0.90*** **	**0.83** **
C	0.35**	0.33**	0.04	0.04	0.20	0.39	0.56**	0.28
DOC	**0.78*** **	0.67***	0.10	0.14	0.30	0.20	0.09	0.09
N	0.29***	0.20*	0.65**	**0.70** **	0.33	0.47*	0.04	0.34
N_{min}	0.17*	0.10	**0.77*** **	0.55*	0.16	0.11	0.18	0.16
N_{org}	0.53***	0.41***	0.13	0.02	0.42*	0.35	0.15	0.26
C: N ratio	0.52***	0.42***	0.68**	0.66**	0.47*	0.42*	0.07	0.28
C: P ratio	0.15*	0.10	0.38	0.40	0.24	0.05	0.54**	0.30
N: P ratio	0.27**	0.24***	0.01	0.11	0.31	0.06	0.53**	0.40*
Ca	0.10	0.05	0.20	0.34	0.02	0.15	0.21	0.41*
Fe	0.46***	0.38***	0.04	0.25	0.07	0.15	0.12	0.03
K	0.07	0.15*	0.07	0.45*	0.21	0.02	0.24	0.23
Mg	0.01	0.24**	0.47*	0.62**	0.27	0.63**	0.44*	0.31
P	0.00	0.02	0.35	0.46*	0.14	0.26	**0.70** **	0.52**

Table S8 Continued

(b) EcM_BL

Factors	All sampling times		0 day		200 days		400 days	
	**Bac**	**Fun**	**Bac**	**Fun**	**Bac**	**Fun**	**Bac**	**Fun**
**Tree factors**								
Tree mycorrhizal type	NA	NA	NA	NA	NA	NA	NA	NA
Tree species	0.02	0.28***	0.60*****	**0.83***	**0.82***	**0.88***	**0.87***	**0.85***
**Time factor**								
Sampling times	**0.81***	**0.86***	NA	NA	NA	NA	NA	NA
**Plot factors**								
Soil water content	0.06	0.05	0.31	0.20	0.25	0.10	0.06	0.07
Soil pH	0.45***	0.38***	0.63**	**0.82**	**0.80***	**0.88***	**0.72**	0.67**
Latitude	0.11	0.14*	0.20	0.21	0.16	0.23	0.14	0.13
Longitude	0.25**	0.33**	0.55*	0.51*	0.35	0.53**	0.33	0.33
**Leaf physicochemical properties**								
Leaf water content	0.14*	0.27**	0.32	0.25	0.35	0.11	0.29	0.17
Leaf pH	0.66***	0.47***	0.38	**0.71**	0.66**	0.64**	0.37	0.20
C	0.45***	0.39***	0.68**	0.61**	0.18	0.24	0.22	0.32
DOC	**0.79***	0.63***	0.42*	0.23	0.47*	0.48*	0.27	0.25
N	0.39***	0.46***	0.47*	0.41*	0.06	0.63**	0.51*	0.35
N_{min}	0.08	0.04	**0.81***	0.63**	0.03	0.04	0.16	0.11
N_{org}	0.43***	0.36**	0.64**	0.54**	0.09	0.13	0.23	0.23
C: N ratio	0.46***	0.50***	0.56*	0.50*	0.06	0.66***	0.51*	0.52*
C: P ratio	0.02	0.01	0.30	0.41*	0.33	0.07	0.44*	0.42
N: P ratio	0.16*	0.20**	0.32	0.59*	0.51**	0.12	0.44*	0.33
Ca	0.24**	0.29**	0.35	0.58**	0.15	0.32	0.54*	0.54*
Fe	0.43***	0.42***	0.15	0.12	0.10	0.07	0.46*	0.42*
K	0.01	0.01	0.43*	0.11	0.38	0.28	0.31	0.43*
Mg	0.21**	0.19*	0.50*	0.48*	0.53*	0.36*	0.12	0.32
P	0.06	0.05	0.19	0.36	0.20	0.21	0.58*	0.33

Table S8 Continued

(c) EcM_C

**Factors**	**All sampling times**		**0 day**		**200 days**		**400 days**	
	**Bac**	**Fun**	**Bac**	**Fun**	**Bac**	**Fun**	**Bac**	**Fun**
**Tree factors**								
Tree mycorrhizal type	NA	NA	NA	NA	NA	NA	NA	NA
Tree species	0.02	0.38***	**0.91*** **	**0.94*** **	0.61**	**1.00*** **	**0.77*** **	**0.97*** **
**Time factor**								
Sampling times	**0.85*** **	**0.79*** **	NA	NA	NA	NA	NA	NA
**Plot factors**								
Soil water content	0.40***	0.18*	0.35	0.29	0.35	0.32	0.21	0.39*
Soil pH	0.22**	0.11	**0.86*** **	**0.90*** **	0.31	**0.85*** **	**0.76** **	**0.87*** **
Latitude	0.20*	0.44***	**0.88** **	**0.94*** **	0.67**	**1.00*** **	0.67**	**0.97*** **
Longitude	0.01	**0.79*** **	**0.92*** **	**0.96*** **	**0.89*** **	**1.00*** **	0.47*	**0.97*** **
**Leaf physicochemical properties**								
Leaf water content	0.14*	0.01	0.15	0.33	0.06	0.03	0.28	0.52**
Leaf pH	0.39***	0.30***	0.51**	0.43*	0.17	0.35	0.32	0.34
C	0.26**	0.26**	**0.76** **	**0.79*** **	0.22	0.69***	0.40*	0.44*
DOC	0.25**	0.24**	**0.83*** **	**0.86*** **	0.61**	0.39	0.47*	0.24
N	0.54***	0.62***	0.47*	0.19	0.69**	0.65**	0.35	**0.82*** **
N_{min}	0.27**	0.27***	0.48**	0.42*	0.37	0.33	0.42*	0.33
N_{org}	0.31**	0.24**	0.32	0.44*	0.16	0.08	0.59**	0.41*
C: N ratio	**0.73*** **	**0.75*** **	0.39	0.14	0.66**	0.58*	0.21	**0.77*** **
C: P ratio	0.19*	0.36***	0.53*	**0.70** **	0.28	0.12	0.25	**0.70** **
N: P ratio	0.38***	0.59***	**0.77** **	**0.88** **	0.42*	0.27	0.29	**0.82*** **
Ca	0.22**	0.20**	0.40*	0.52**	0.12	0.52*	**0.70*** **	0.68***
Fe	0.29***	0.30***	0.14	0.18	0.06	0.07	0.18	0.02
K	0.42***	0.51***	0.28	0.52**	**0.71** **	0.63**	0.27	0.33
Mg	0.12	0.10	0.04	0.02	0.04	0.02	0.18	0.29
P	0.26**	0.39***	0.62**	0.80**	0.30	0.30	0.29	**0.77*** **

Table S9 Goodness-of-fit statistics (R^2) of enzyme activities fitted to the non-metric multidimensional scaling (NMDS) ordination of bacterial and fungal communities in all tree mycorrhizal types, AM_BL tree species, EcM_BL, and EcM_C based on relative abundance data and the Bray–Curtis distance measure. Bold letters indicate statistical significance with $R^2 \geq 0.70$. * P<0.05, ** P<0.01, *** P<0.001.

Enzyme activities	All sampling times		200 days		400 days	
	Bac	Fun	Bac	Fun	Bac	Fun
**All tree mycorrhizal types**						
β-glucosidase	0.47***	0.23***	0.39***	0.28**	0.64***	0.67***
N-acetylglucosaminidase	0.29***	0.10**	0.2**	0.06	0.23**	0.23**
Acid phosphatase	0.30***	0.40***	0.44***	0.34***	0.55***	0.63***
General peroxidase	0.35***	0.07*	0.10	0.17*	0.01	0.12
Manganese peroxidase	0.32***	0.03	0.05	0.10	0.20*	0.14*
**AM_BL tree species**						
β-glucosidase	0.63***	0.04	0.55*	0.68**	**0.79*** **	**0.78*** **
N-acetylglucosaminidase	**0.71*** **	**0.75*** **	**0.76*** **	**0.87*** **	**0.83*** **	**0.89*** **
Acid phosphatase	**0.77*** **	0.65***	**0.75*** **	**0.86*** **	**0.83*** **	**0.95*** **
General peroxidase	0.66***	0.40***	0.10	0.55*	**0.80*** **	**0.92*** **
Manganese peroxidase	0.56***	0.26**	0.17	0.46*	**0.84*** **	**0.95*** **
**EcM_BL tree species**						
β-glucosidase	0.68***	0.12	**0.93*** **	**0.92*** **	0.65**	**0.85*** **
N-acetylglucosaminidase	0.64***	**0.75*** ***	0.66***	**0.86*** **	**0.90*** **	**0.85*** **
Acid phosphatase	0.33**	0.33**	0.37	**0.87*** **	0.65**	**0.85*** **
General peroxidase	**0.81*** **	0.01	0.28	**0.83*** **	0.67***	**0.83*** **
Manganese peroxidase	0.66***	0.18	0.25	**0.84*** **	**0.90*** **	**0.85*** **
**EcM_C tree species**						
β-glucosidase	0.30*	0.32**	0.30	**0.85*** **	**0.74** **	**0.97*** **
N-acetylglucosaminidase	0.53***	0.13	0.41	**0.99*** **	**0.88*** **	**0.98*** **
Acid phosphatase	**0.73*** **	0.42**	0.28	**0.90*** **	0.60**	**0.97** **
General peroxidase	0.61***	0.34**	0.28	**0.92*** **	**0.80*** **	**0.98*** **
Manganese peroxidase	**0.76*** **	0.18	0.28	**0.98*** **	0.62**	**0.97*** **

Figure S1. Composition of the bacterial community (at class-level (a) and genus-level (b), considering only classes and genera with relative sequence read abundances ≥ 1%, the rest of the bacterial classes and genera were pooled as “others”) associated with leaf and needle decomposition based on relative abundance. The abbreviations are AM_BL: broadleaved arbuscular mycorrhizal trees (including AH: *Acer pseudoplatanus*, ES: *Fraxinus excelsior*, and KB: *Prunus avium*), EcM_BL: broadleaved ectomycorrhizal trees (including BU: *Fagus sylvatica*, HBU: *Carpinus betulus*, and LI: *Tilia cordata*), and EcM_C: coniferous ectomycorrhizal trees (including FI: *Picea abies*, KI: *Pinus sylvestris*, and DG: *Pseudotsuga menziesii*).

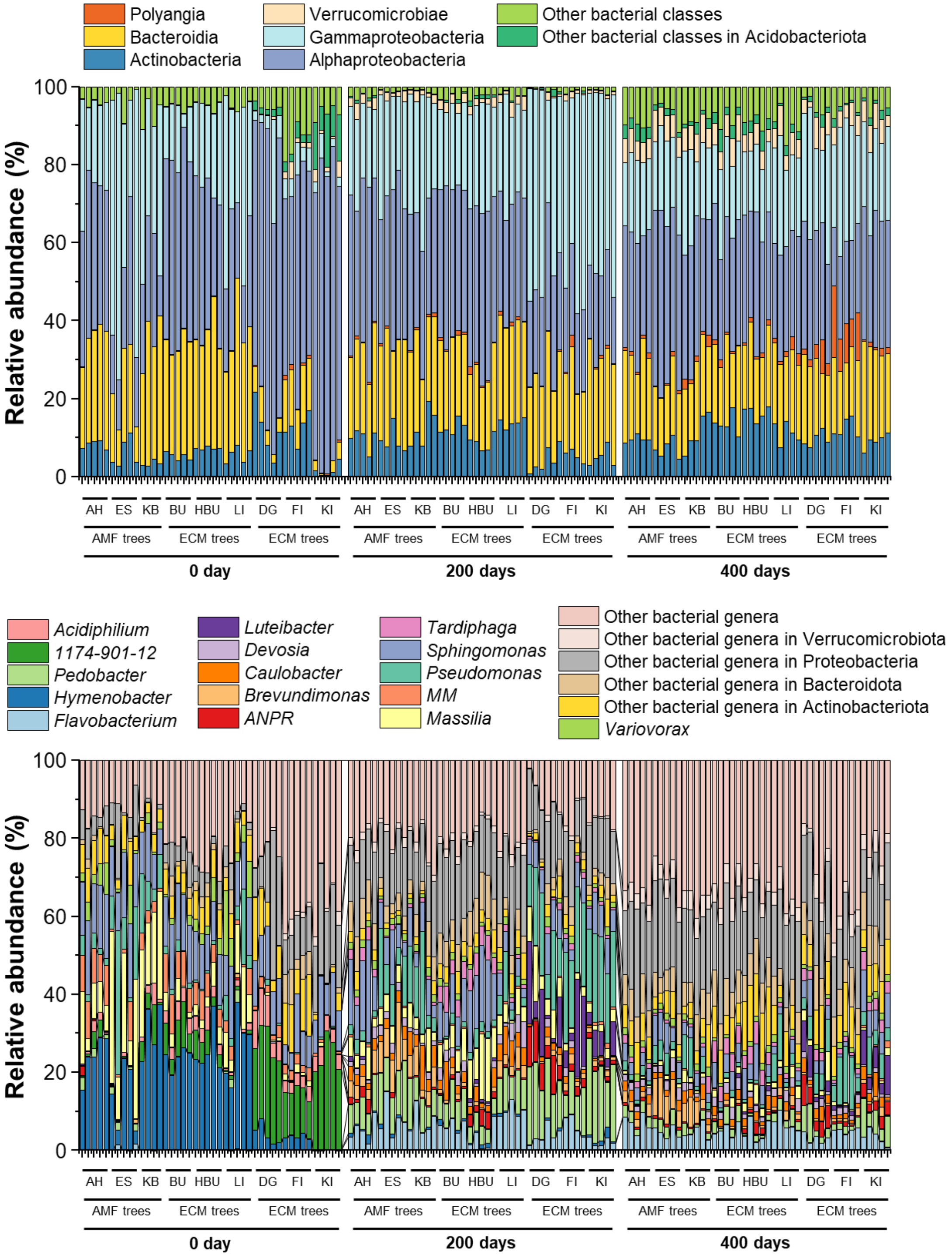

Figure S2 Top-50 bacterial ASVs with the highest relative abundance among all treatments. Treatment abbreviations are AM_BL: broadleaved arbuscular mycorrhizal trees (including AH: *Acer pseudoplatanus*, ES: *Fraxinus excelsior*, and KB: *Prunus avium*), EcM_BL: broadleaved ectomycorrhizal trees (including BU: *Fagus sylvatica*, HBU: *Carpinus betulus*, and LI: *Tilia cordata*), and EcM_C: coniferous ectomycorrhizal trees (including FI: *Picea abies*, KI: *Pinus sylvestris*, and DG: *Pseudotsuga menziesii*).

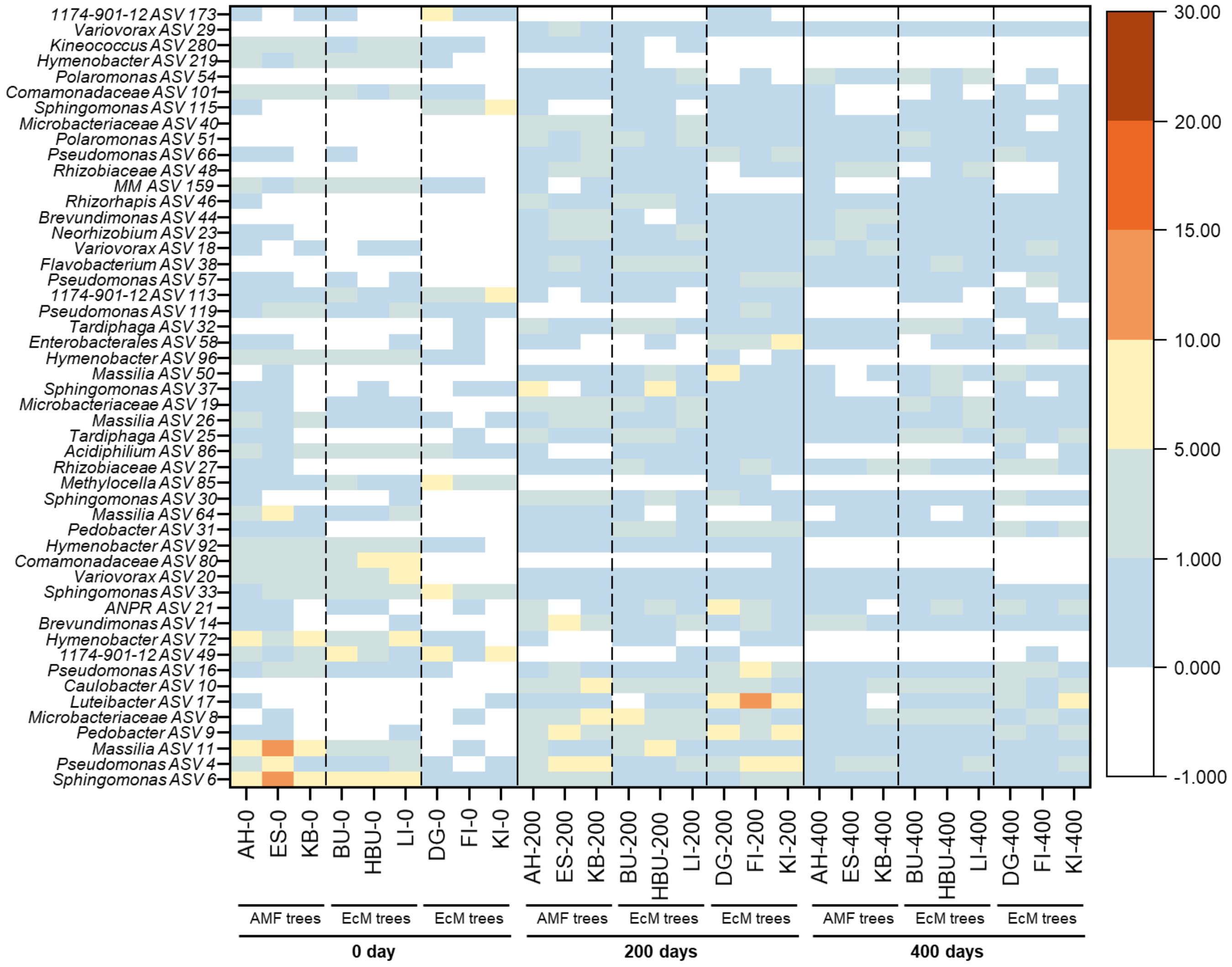

Figure S3. The composition of the fungal community (at class-level (a) and genus-level (b), considering only classes and genera with relative sequence read abundances ≥ 1%, the rest of the fungal classes and genera were pooled as “others”) associated with leaf and needle decomposition based on relative abundance. The abbreviations used are shown in Fig. 1.

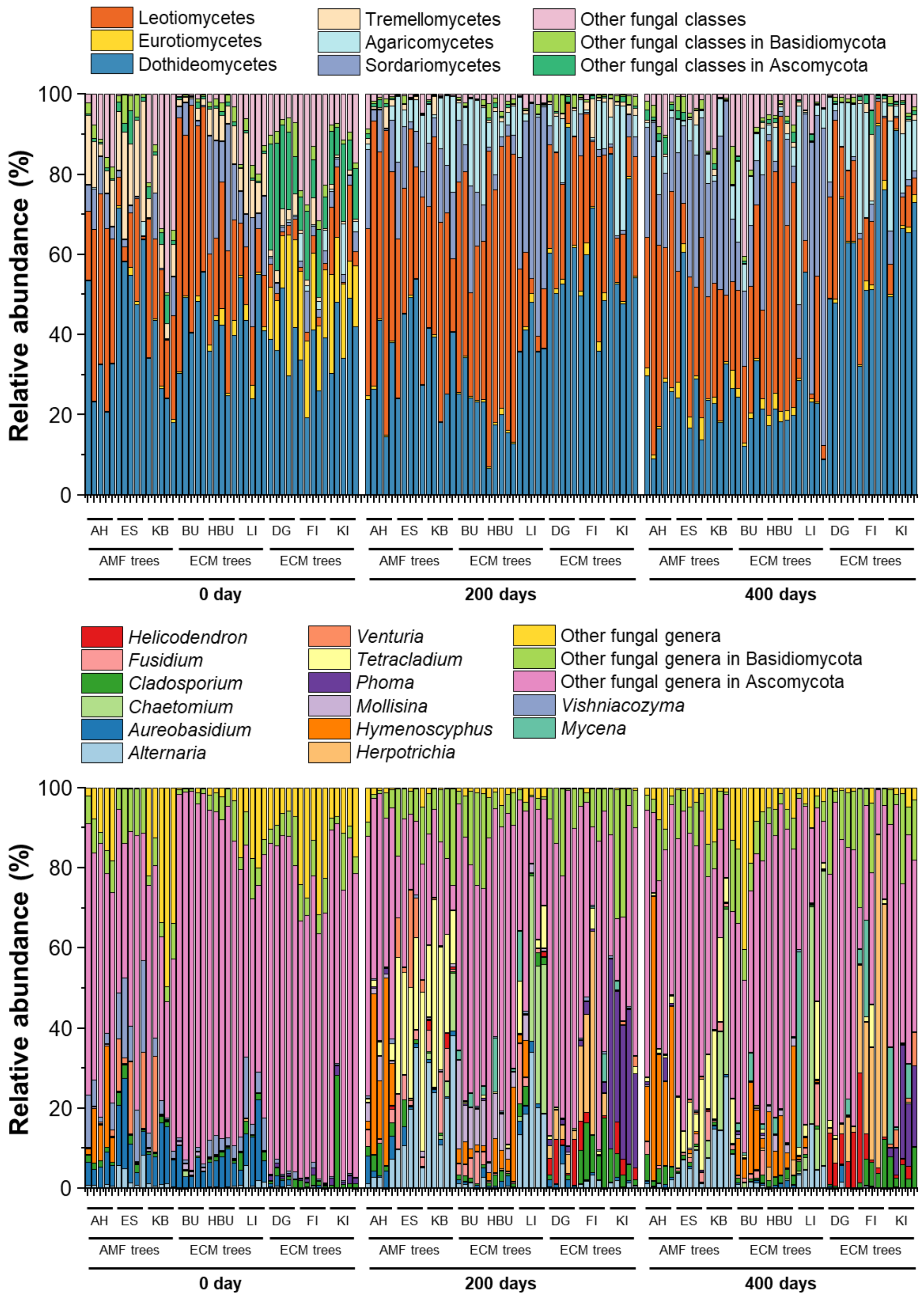

Figure S4 Top-50 fungal ASVs with the highest relative abundance across all treatments. The details of the treatment abbreviations can be found in the legend of Fig. 1.

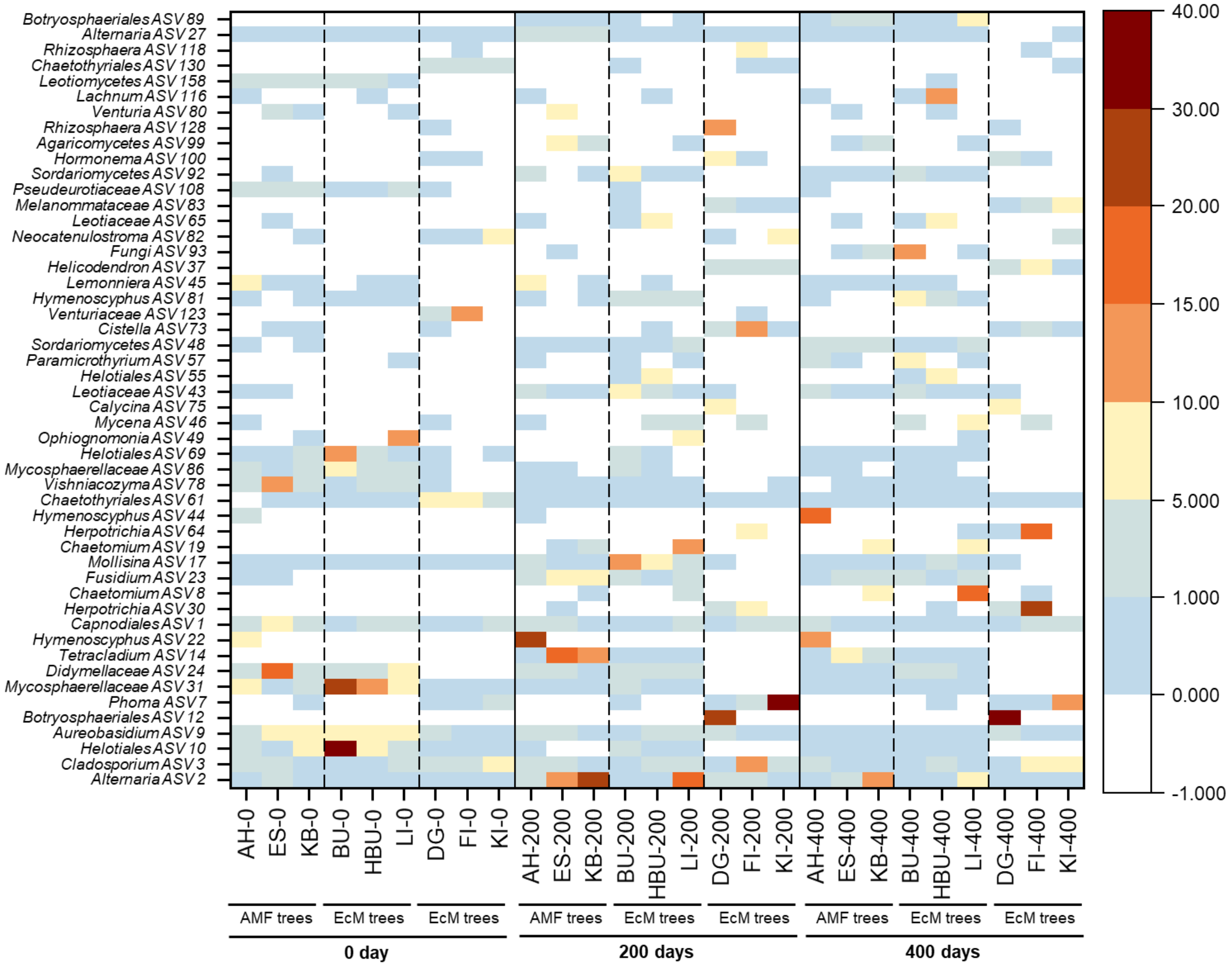

Figure S5. Hydrolytic (β-glucosidase, N-acetylglucosaminidase, and acid phosphatase) and oxidative (PO: general peroxidase and PER: manganese peroxidase) enzyme activities in decomposing leaves and needles from 200 and 400 days. Results are expressed in nmol hr^{-1} g dry $leaf^{-1}$. Details of the treatment abbreviations are provided in the legend of Fig. 1.

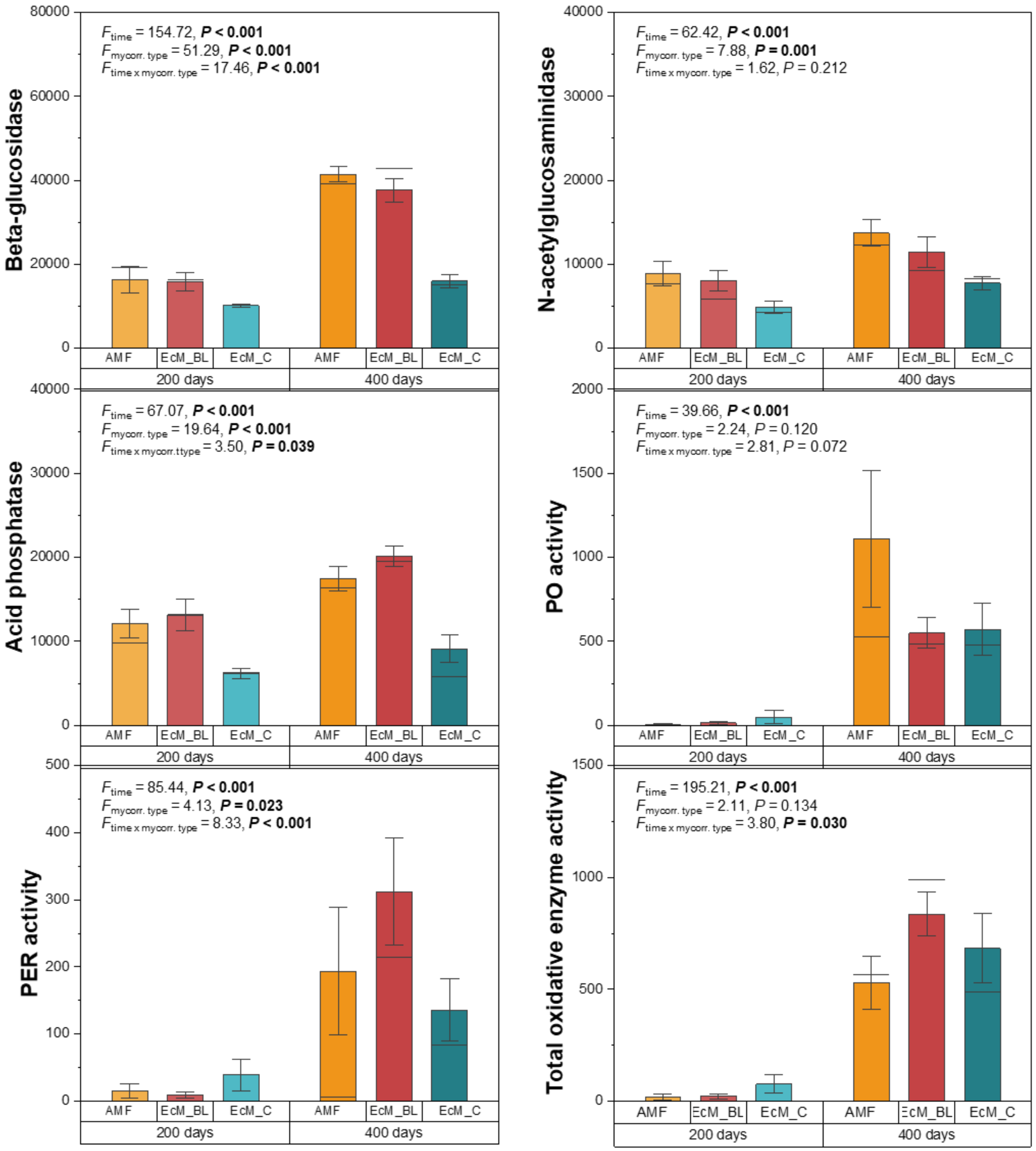

Supplement: Supplementary file 1 [file DataSheet_1.zip › Supplementary Figures and Tables.pdf]
